# Supplementary material for: The cotranslational cycle of the ribosome-bound Hsp70 homolog Ssb
Source: Nat Commun. 2026 Jan 16;17:961. doi: 10.1038/s41467-025-67685-6 (PMC12847954; doi:10.1038/s41467-025-67685-6)
Supplement: Supplementary file 1 — Supplementary Information [file 41467_2025_67685_MOESM1_ESM.pdf]

The cotranslational cycle of the ribosome-bound Hsp70 homolog Ssb

Ying Zhang[1*], Lorenz Grundmann[2,3*], Leonie Vollmar[4,5], Julia Schimpf[4,5], Volker Hübscher[1], Mohd Areeb[1,5], Irina Grishkovskaya[2], Anna Moddemann[1], Kerstin Werner[1], Thorsten Hugel[4,6], David Haselbach[2], and Sabine Rospert[1,6]

Supplementary Data

Supplementary Figures

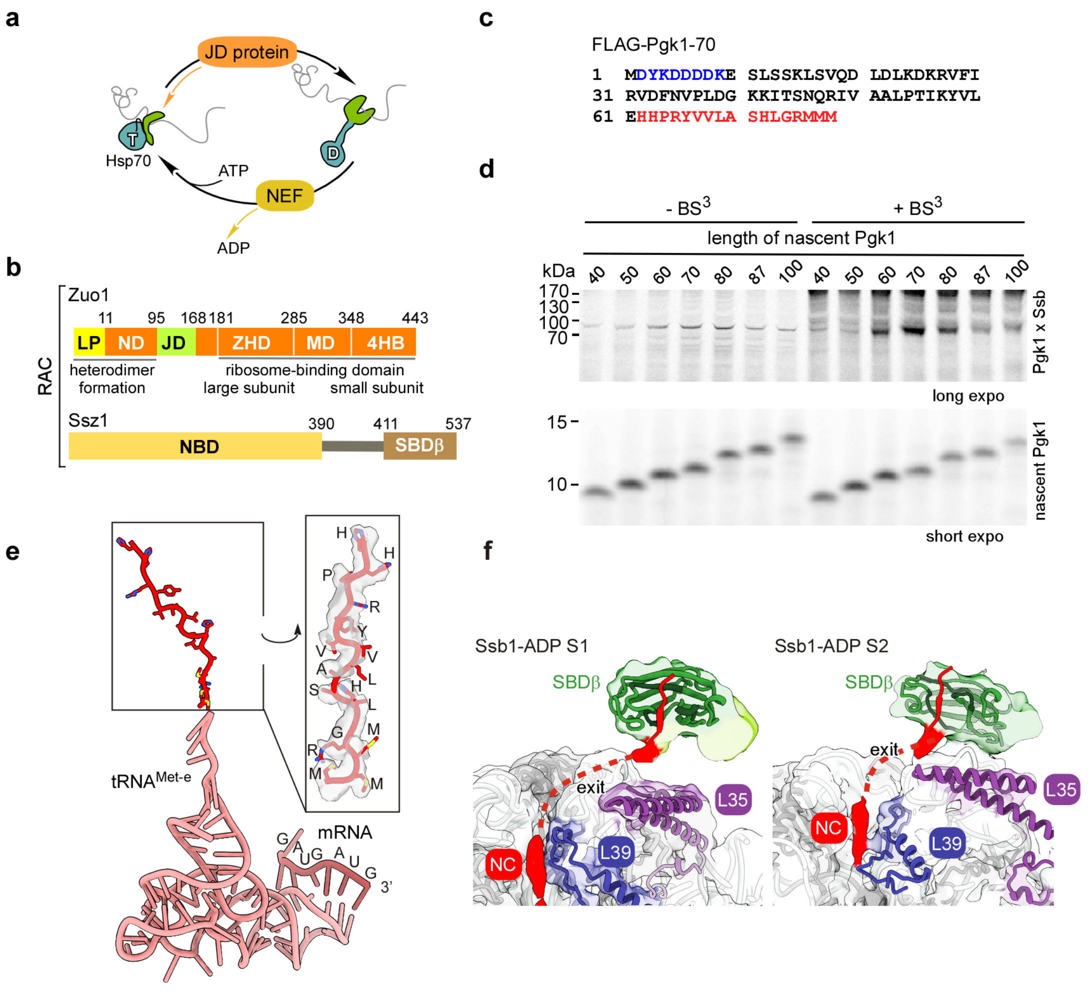

Supplementary Fig. 1. The RAC-Ssb chaperone system and nascent FLAG-Pgk1-70. (a) Basic Hsp70 chaperone cycle. Schematic representation of Hsp70 in the ATP-bound (T) open conformation with low affinity for substrate proteins (gray) and Hsp70 in the ADP-bound closed conformation (D) with high affinity for substrate proteins. ATP hydrolysis is triggered by transient interaction with J-domain (JD) cochaperones. ADP release and rebinding of ATP is stimulated by cochaperones, which act as nucleotide exchange factors (NEFs) [1,2]. **(b)** Domain

structure of the heterodimeric ribosome-associated complex (RAC) consisting of the JD protein Zuo1 and the non-canonical Hsp70 homolog Ssz1 [3]. Zuo1 N-domain (ND, orange) LP-motif (LP, yellow), J-domain (JD, limon), zuotin homology domain (ZHD, orange), middle domain (MD, orange), 4-helix bundle (4HB, orange), Ssz1-NBD (yellow orange), Ssz1-SBDβ (sand), Ssz1 linker region (gray). For details see Introduction. **(c)** N-terminal sequence of FLAG-tagged 3-phosphoglycerate kinase. The FLAG-tag of FLAG-Pgk1-70 is indicated in blue, nascent chain residues resolved in the cryo-EM maps Ssb-ADP S1 and S2 are shown in red. **(d)** Nascent FLAG-Pgk1-70 efficiently crosslinks to Ssb. RNCs carrying 40 to 100 residues of radiolabeled nascent Pgk1 (length is given omitting the FLAG-tag) were incubated in the presence or absence of the crosslinker BS^3 as described in Methods. Presented are two exposures of the same gel, which show nascent Pgk1 (short exposure) or crosslink products between nascent Pgk1 and Ssb (Pgk1 x Ssb, long exposure). **(e)** Nascent FLAG-Pgk1-70 is attached to RNCs via $tRNA^{Met\text{-}e}$. Atomic model of $tRNA^{Met\text{-}e}$ (salmon) attached to nascent FLAG-Pgk1-70 (red) and the 3´-prime end of the truncated mRNA (firebrick) of Ssb-ADP S2. The inset shows an overlay of the post-processed cryo-EM density of the Ssb-ADP S2 and the atomic model of the nascent chain. **(f)** Binding of the nascent chain to the peptide-binding cleft of Ssb-SBDβ. Overlays of cross sections through gaussian filtered (sdev=2.5), non-post-processed cyro-EM maps and corresponding atomic models of Ssb-ADP S1 and S2. The nascent chain model was placed by aligning PDB 1DKZ [4] into Ssb-SBDβ of Ssb-ADP S1 and S2.

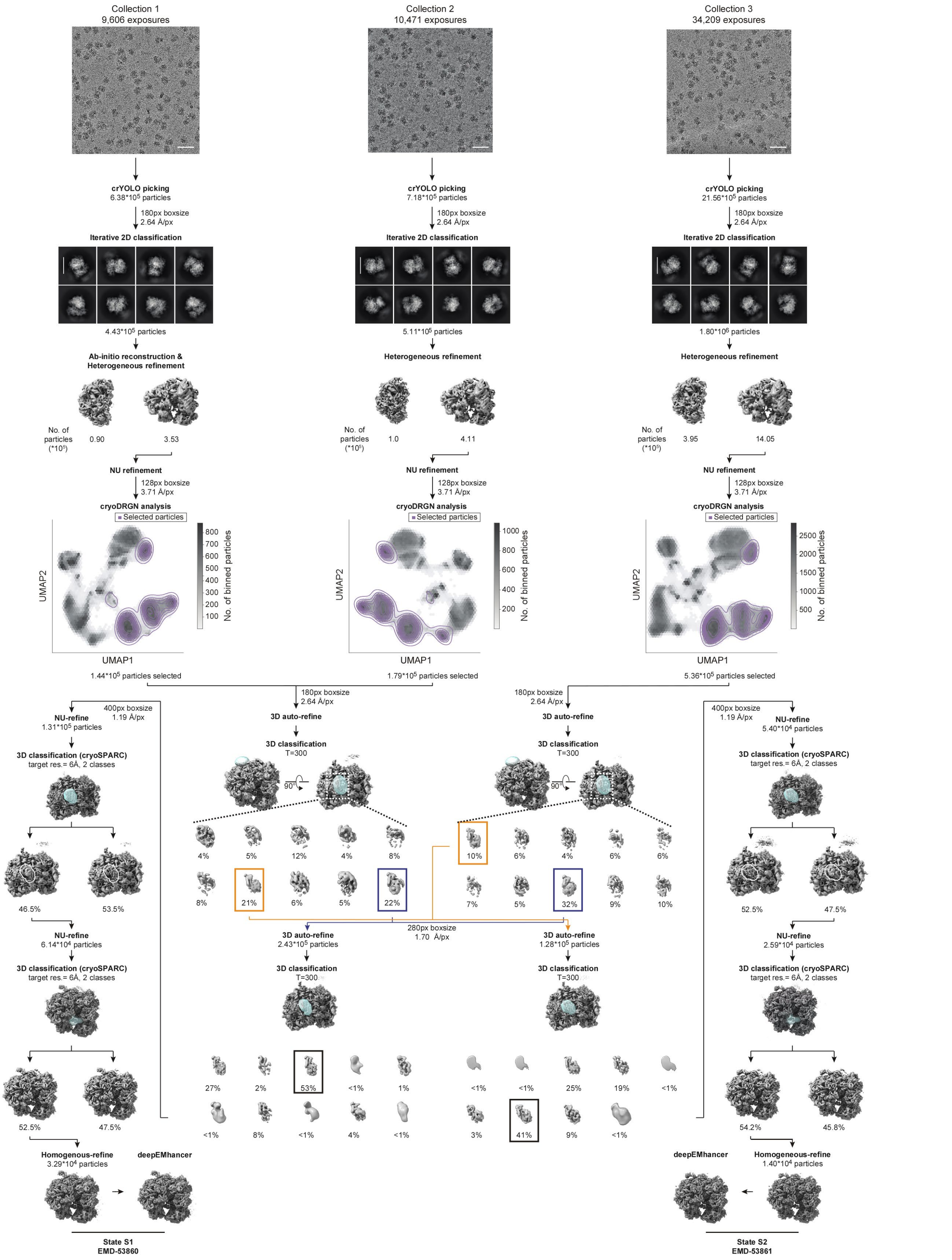

Supplementary Fig. 2. Cryo-EM processing workflow. For a detailed description of cryo-EM processing see Methods.

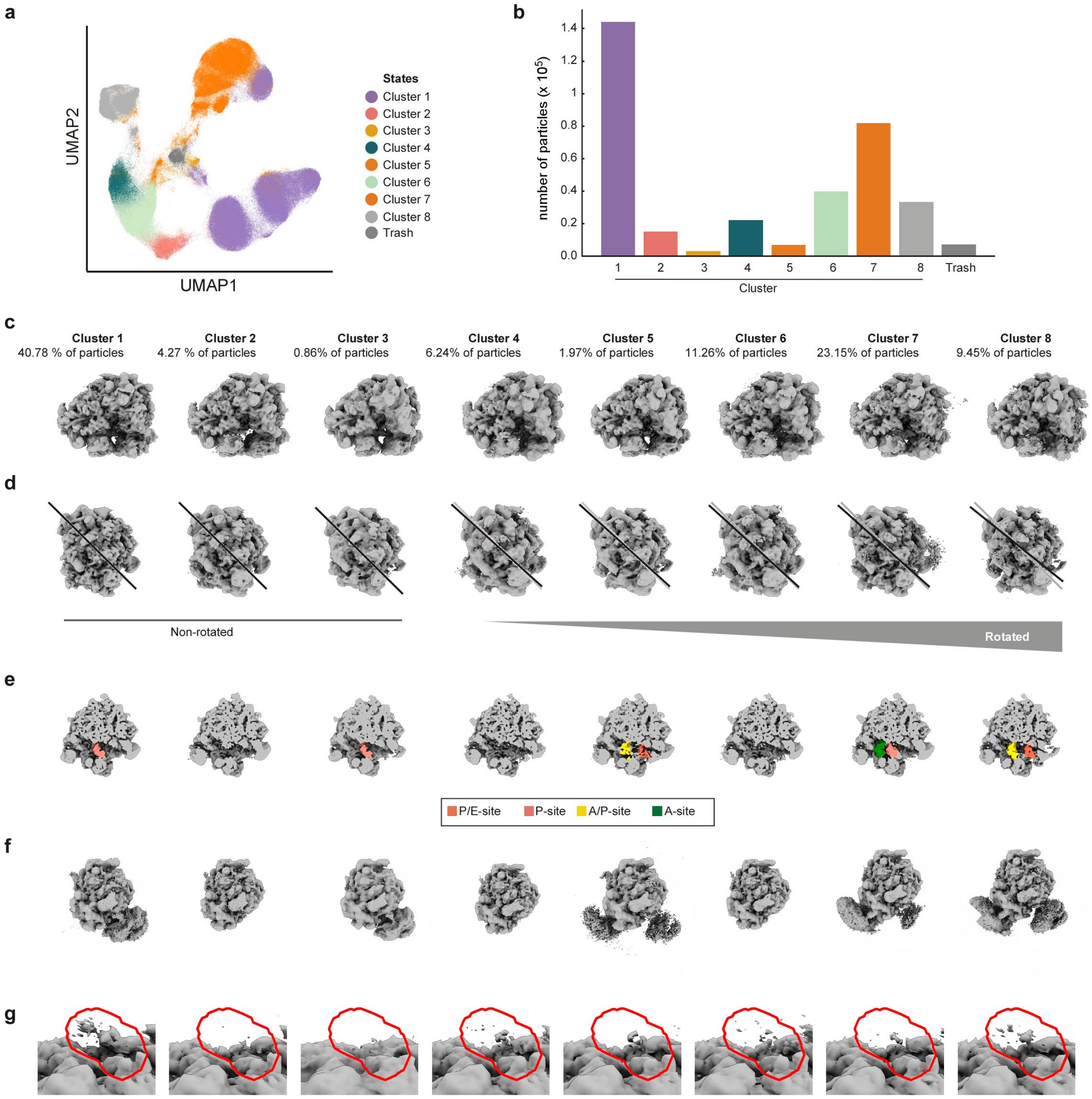

Supplementary Fig. 3. Landscape analysis of translational states of RNCs. (**a**) 2D representation of the 8-dimensional cryoDRGN enconded latent space. Dimensionality reduction was performed by UMAP. Clustering results are colored as indicated. (**b**) Particle distribution across clusters 1 to 8. Color code as in **a**. (**c**) cryoDRGN reconstruction of particles in clusters 1 to 8. The number of particles in each cluster is indicated as a percentage. (**d**) Rotational state of the 40S ribosomal subunit in clusters 1 to 8. The axes connect the tip of expansion segment 6 (ES6) of the 18S rRNA to the C-terminus of the 40S ribosomal protein Asc1. Non-rotated axis (black line), rotated axis (gray line), with the 60S subunit held fixed. (**e**) tRNA states in cross sections through reconstituted maps. P/E-site tRNA (orange), P-site tRNA (salmon), A/P-site tRNA (yellow), and A-site tRNA (green). (**f**) Polysome configuration of clusters 1 to 8. Shown are low-pass filtered maps (sdev = 1.5). (**g**) Close-up of Ssb-SBD density at the ribosomal tunnel exit in clusters 1 to 8 from Collection 1 (Supplementary Fig. 2). The Ssb binding region is highlighted with a red outline. Shown are the representative results of Collection 1 (Supplementary Fig. 2). For details see Methods.

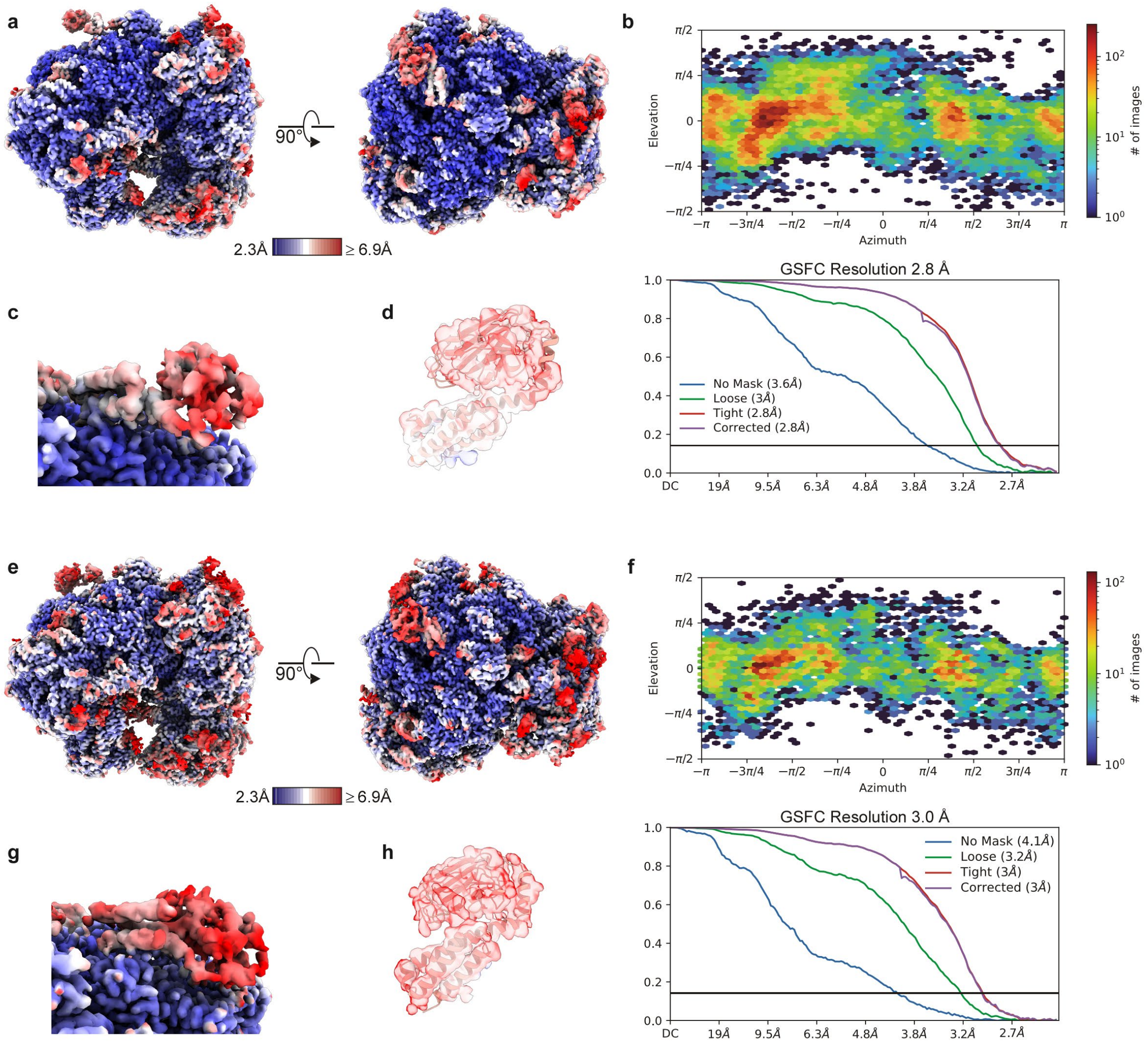

Supplementary Fig. 4. Cryo-EM reconstruction of Ssb bound to 80S ribosomes. (a) Cryo-EM reconstruction of Ssb bound to 80S ribosomes. cryo-EM reconstruction of the S1 state (Ssb-ADP S1) Ssb bound ribosome colored by local resolution. **(b)** Corresponding gold standard Fourier shell correlation plot (GSFSC-plot) for global resolution estimation, viewing angle distribution plot and **(c)** close up of the cryo-EM reconstruction for Ssb (S1 state). **(d)** Fit of the molecular model of Ssb (S1 state) with the corresponding reconstruction, transparently colored by local resolution. **(e)** cryo-EM reconstruction of the S2 state (Ssb-ADP S2) Ssb bound ribosome colored by local resolution. **(f)** Corresponding GSFSC-plot for global resolution estimation, viewing angle distribution plot and **(g)** close up of the cryo-EM reconstruction for Ssb (S2 state). **(h)** Fit of the molecular model of Ssb (S2 state) with the corresponding reconstruction, transparently colored by local resolution.

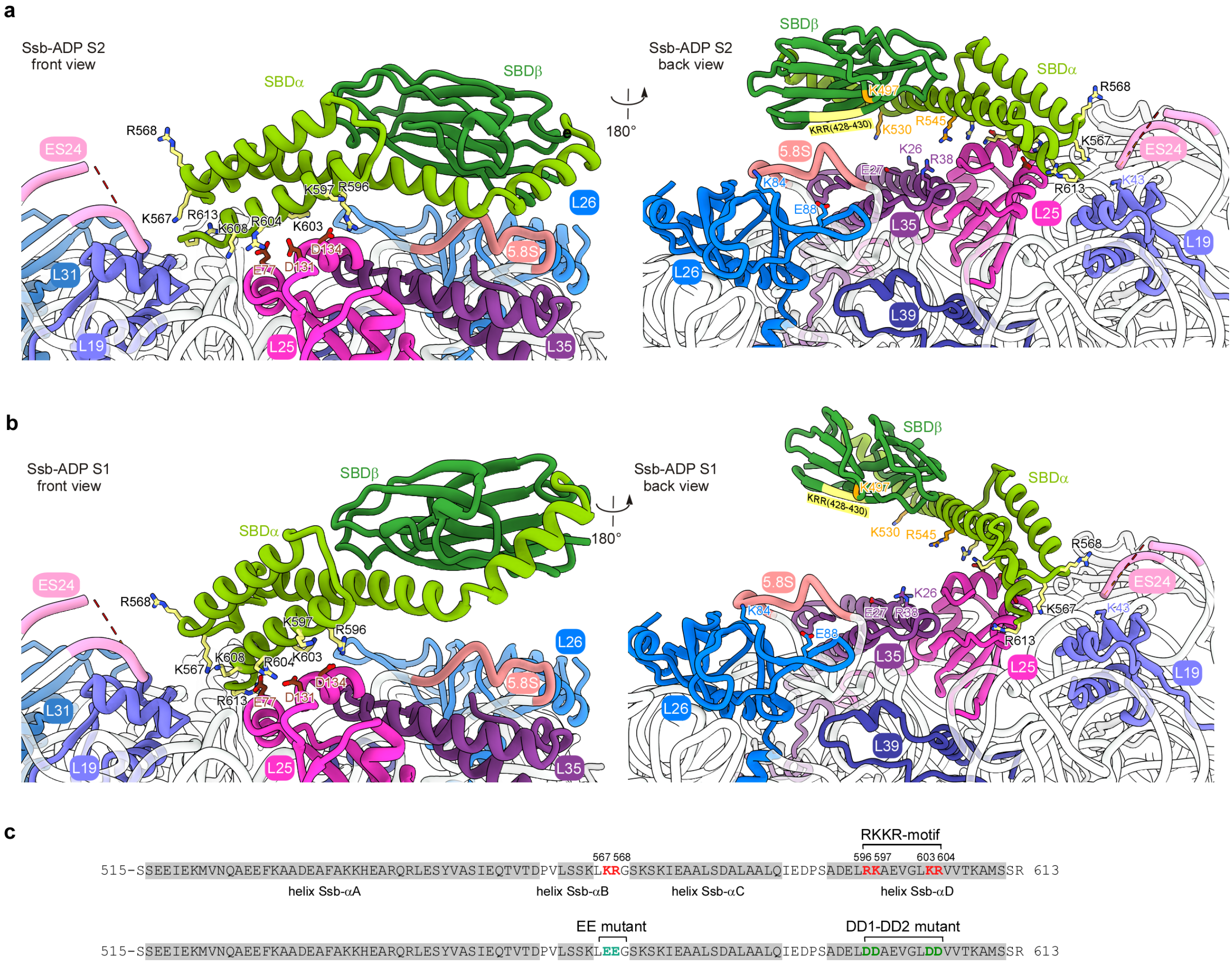

Supplementary Fig. 5. Ribosomal contacts of Ssb. (a) Contacts of Ssb with the ribosomal surface in the atomic model Ssb-ADP S2. The RKKR-motif in helix Ssb-αD interacts with the EDD-motif of Rpl25, and Ssb-K567/Ssb-R568 contact the tip of 25S rRNA h59-ES24 (front view). Furthermore, residues within helix Ssb-αA of SBDα and Ssb-SBDβ are close to the ribosomal surface and within crosslinking distance of Rpl35, Rpl19, and Rpl26. Ssb-K530 is 8 Å from Rpl35-K26 and Ssb-K567 is 6 Å from Rpl19-K43 (back view), consistent with *in vitro* NH_2-specific crosslinking [5]. Ssb-R545 forms *in vivo* site-specific crosslinks with Rpl35, Ssb-K497 with Rpl26, and Rpl35-E27/K26/R38 as well as Rpl26-K84/E88 form *in vivo* site-specific crosslinks with Ssb [6] (back view). Residues 428-430 (KRR-motif) in Ssb-SBDβ contact 5.8S rRNA nucleotides UUC (81-83) (back view), providing a rationale for the moderately reduced ribosome-binding of Ssb mutants in which the KRR motif was replaced with alanine residues [7]. For details, refer to the Results and Supplementary Note 1. **(b)** Contacts of Ssb with the ribosomal surface in the atomic model of Ssb-ADP S1. Similar to the S2 state shown in **a**, the RKKR-motif of Ssb-ADP S1 interacts with the EDD-motif of Rpl25, and Ssb-K567/Ssb-R568 are close to h59-ES24 (front view). However, helix Ssb-αA of SBDα and Ssb-SBDβ are positioned farther from the ribosomal surface, with distances of 16 Å between Ssb-K530 and Rpl35-K26, and 11 Å between Ssb-K567 and Rpl19-K43 (back view). For details, refer to the Results and Supplementary Note 1. Color code in **a** and **b**: Ssb-SBDβ (forest), Ssb-SBDα, (split pea), Ssb residues involved in ribosome-binding (yellow), Ssb residues involved in site specific crosslinking (brightorange), Rpl25 residues involved in ribosome-binding of Ssb (chocolate), Rpl25 (light magenta), Rpl35 (violetpurple), Rpl26 (marine), Rpl19 (slate blue), Rpl31 (sky blue), Rpl39 (deepblue), helix 59 expansion segment 24 (ES24, pink), 5.8S rRNA (5.8 S, salmon). **(c)** Amino acid sequence of Ssb-SBDα and mutations within Ssb-SBDα affecting ribosome-binding of Ssb. Upper sequence: Ssb-SBDα consisting of the 4 most C-terminal α-helices, termed Ssb-αA, Ssb-αB, Ssb-αC, and Ssb-αD [5]. Lower sequence: amino acids replaced in Ssb-DD1-DD2 (this work) or Ssb-EE/L_{BC} [5] are highlighted in green. For details see Results and Supplementary Note 1.

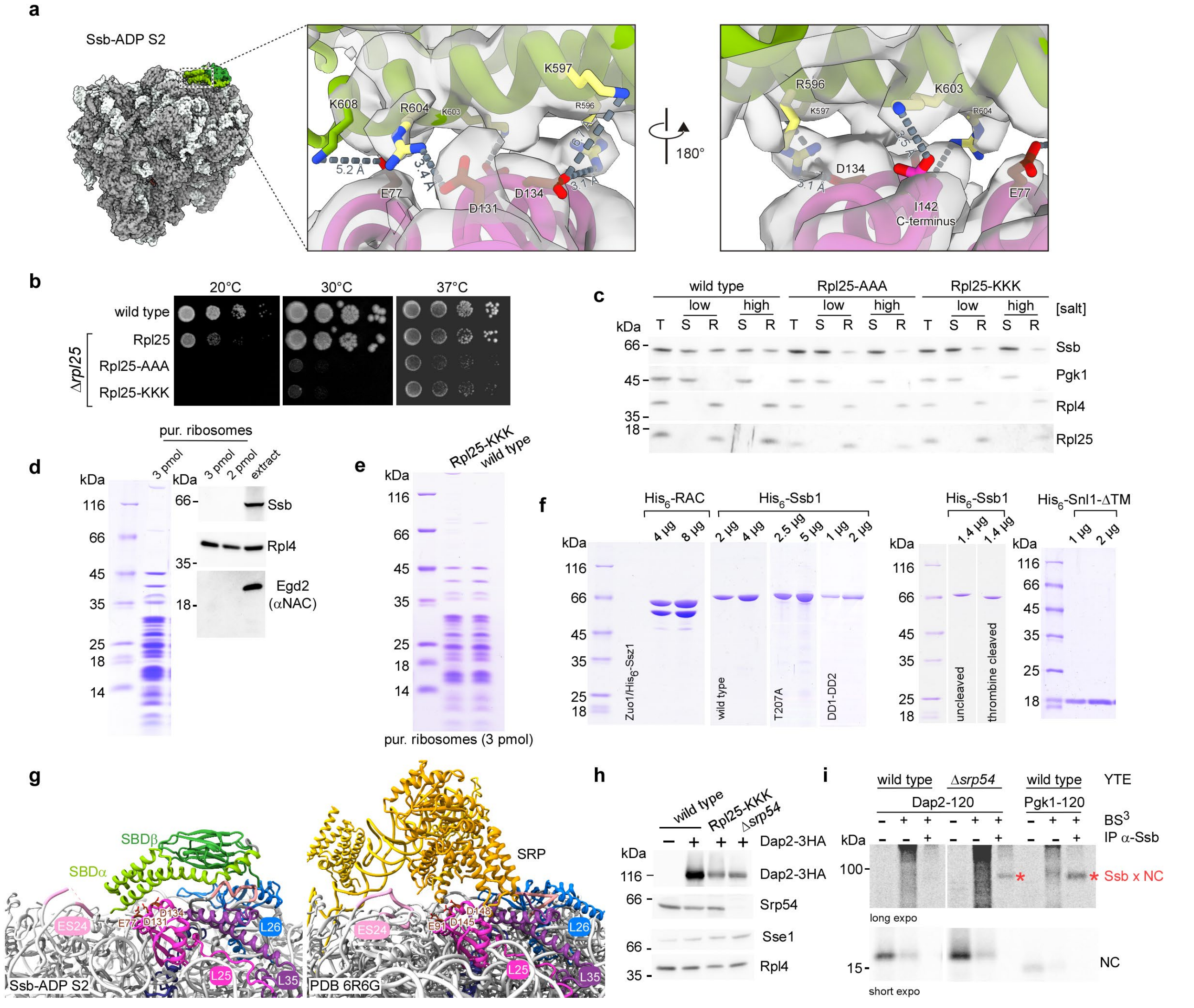

Supplementary Fig. 6. The interaction between Ssb and Rpl25 is essential for efficient ribosome-binding of Ssb. (a) The RKKR-motif within helix Ssb-αD contacts the EDD-motif within Rpl25. DeepEMhanced cryo-EM map of Ssb-ADP S2 and close-ups of the contact zone depicting overlays of cryo-EM map and atomic model. Residues of the RKKR-motif (yellow, Supplementary Fig. 5c), and Rpl25 (light magenta) residues of the EDD-motif within Rpl25 (chocolate). Molecular interactions and corresponding distances are depicted in dark gray. **(b)** Strains carrying mutations in the EDD-motif of Rpl25 display severe growth defects. Serial 10-fold dilutions of log-phase wild type or Δ*rpl25* cells complemented with plasmid encoded Rpl25, Rpl25-AAA, or Rpl25-KKK were spotted onto YPD plates and were incubated at the indicated temperatures for 3 days. **(c)** Mutations within the Rpl25 EDD-motif significantly reduce ribosome-binding of Ssb. Ribosome-binding assays were performed with total cell extract (T) derived from wild type, Rpl25-AAA, or Rpl25-KKK cells in low-salt (120 mM KOAc) or high-salt (800 mM KOAc) conditions. The cytosolic supernatant (S) was separated from ribosomal pellets (R) by centrifugation through sucrose cushions and aliquots were subsequently analyzed by immunoblotting with antibodies directed against the proteins indicated. Statistical analysis is shown in Fig. 2b. **(d)** Purified non-translating wild type ribosomes are free of Ssb and nascent polypeptide associated complex (NAC). Purification of ribosomes was performed as described in Methods. Shown is a Coomassie-stained gel and immunoblots decorated with the antibodies indicated. Egd2 is the α-subunit of heterodimeric NAC. Total cell extract was loaded as a control. **(e)** Side by side analysis of purified non-translating wild type and Rpl25-KKK ribosomes. Shown is a Coomassie-stained gel. **(f)** Purified proteins employed for cryo-EM, ribosome-binding and crosslinking experiments. His_6-Ssb1 was compared side by side with His_6-Ssb1 after thrombin cleavage removing the His_6-tag. Shown are Coomassie-stained gels. For details on the purification procedures see Methods. **(g)** Ssb and SRP possess overlapping ribosomal binding sites. Side by side view of contacts between Ssb-ADP S2 and Rpl25 and contacts between mammalian SRP and Rpl25 (PDB 6R6G, [8]). The Rpl25 EDD-motif is highly conserved

between yeast and mammals: yeast Rpl25-E77-D131-D134 corresponds to rabbit Rpl25-E91-D145-D148. The color code is as in Supplementary Fig. 5a,b. **(h)** The steady state expression level of the type II ER membrane protein Dap2 is strongly reduced the Rpl25-KKK cells. Yeast lysate prepared from wild type, Rpl25-KKK, and Δ*srp54* cells expressing plasmid encoded Dap2-3HA were prepared as described [9]. Samples were analyzed by immunoblotting using the indicated antibodies; Dap2-3HA was detected with α-HA. **(i)** Ssb interacts with the SRP-substrate Dap2 only when SRP is absent. RNCs carrying radiolabeled nascent Dap2-120 or Pgk1-120 were crosslinked with BS^3. Crosslinks between Ssb and nascent Dap2-120 or Pgk1-120 were isolated by immunoprecipitation (IP) with α-Ssb. Shown is a long and a short exposure of the same autoradiograph. Nascent chain (NC).

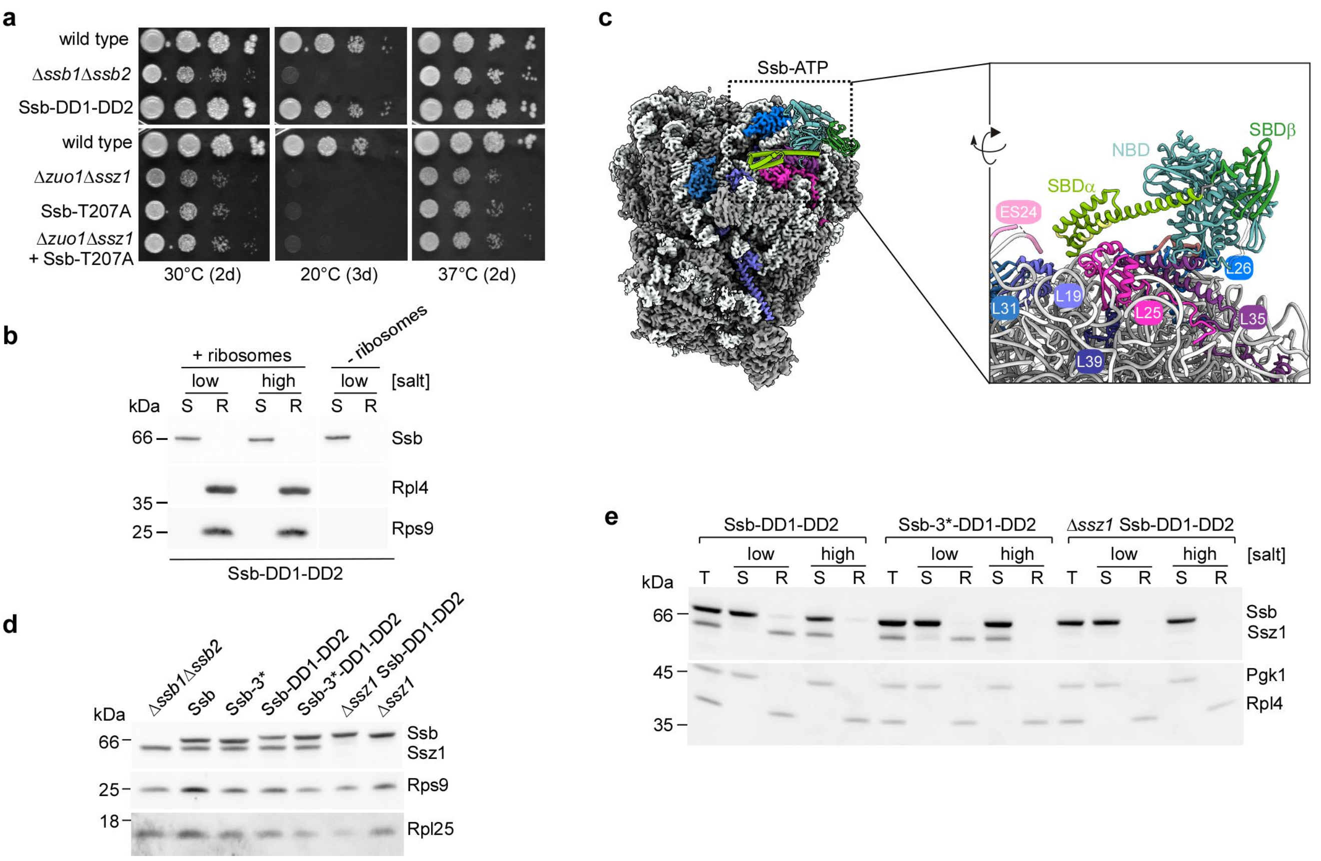

Supplementary Fig. 7. Model of ribosome-bound Ssb in the ATP-bound state. (a) Growth analysis of strains expressing genomically encoded Ssb-DD1-DD2 and Ssb2-DD1-DD2 in a wild type background or genomically encoded Ssb-T207A and Ssb2-T207A in a wild type or Δ*zuo1*Δ*ssz1* background. Serial 10-fold dilutions of logarithmically growing cells were spotted onto YPD plates, which were incubated for the indicated times at the indicated temperatures. **(b)** Ribosome-binding of Ssb-DD1-DD2 to purified wild type ribosomes is strongly reduced. Ribosome-binding assays were performed with purified components as in Fig. 2c. Supernatants (S) and ribosomal pellets (R) were analyzed by immunoblotting with antibodies directed against the indicated proteins. **(c)** Model of ribosome-bound Ssb-ATP. The SBDα domain of Ssb-ATP (*S.c.* Ssb-ATP, see Methods) was docked into the cryo-EM density map of Ssb-ADP in state S1, such that the position of SBDα was congruent. This positioning of Ssb-ATP is consistent with the absence of crosslinks between Ssb-ATP and Rpl35, Rpl19, Rpl39, or Rpl26 (see also Supplementary Fig. 9a, Fig. 5). The inset represents a side view of the Ssb-ATP model. Ssb-SBDβ (forest), Ssb-SBDα (split pea), Ssb-NBD (light teal), and residues of the RKKR-motif, which contact Rpl25 and K567/R568, which contact the tip of ES24 are highlighted in yellow. The color code of ribosomal proteins and rRNA is detailed in Supplementary Fig. 5a,b. **(d)** Ssb mutants with ribosome-binding defects are expressed at wild type levels. Expression level of wild type Ssb1 and of the indicated mutant Ssb1 variants expressed from low-copy plasmids (Supplementary Table 3). Immunoblots were analyzed with the indicated antibodies. **(e)** Synthetic disruption of ribosome-binding caused by a combination of Ssb mutations affecting direct ribosome-binding (Ssb-DD1-DD2) and indirect ribosome-binding (Ssb-3*) mediated by Ssz1. Shown is the fraction of Ssb co-sedimenting with ribosomes in total cell extract derived from Δ*ssb1*Δ*ssb2* expressing Ssb-DD1-DD2, Ssb-3*-DD1-DD2, or Δ*ssz1*Δ*ssb1*Δ*ssb2* Ssb-3*-DD1-DD2 as shown in **d**. Statistical analysis is shown in Fig. 3h.

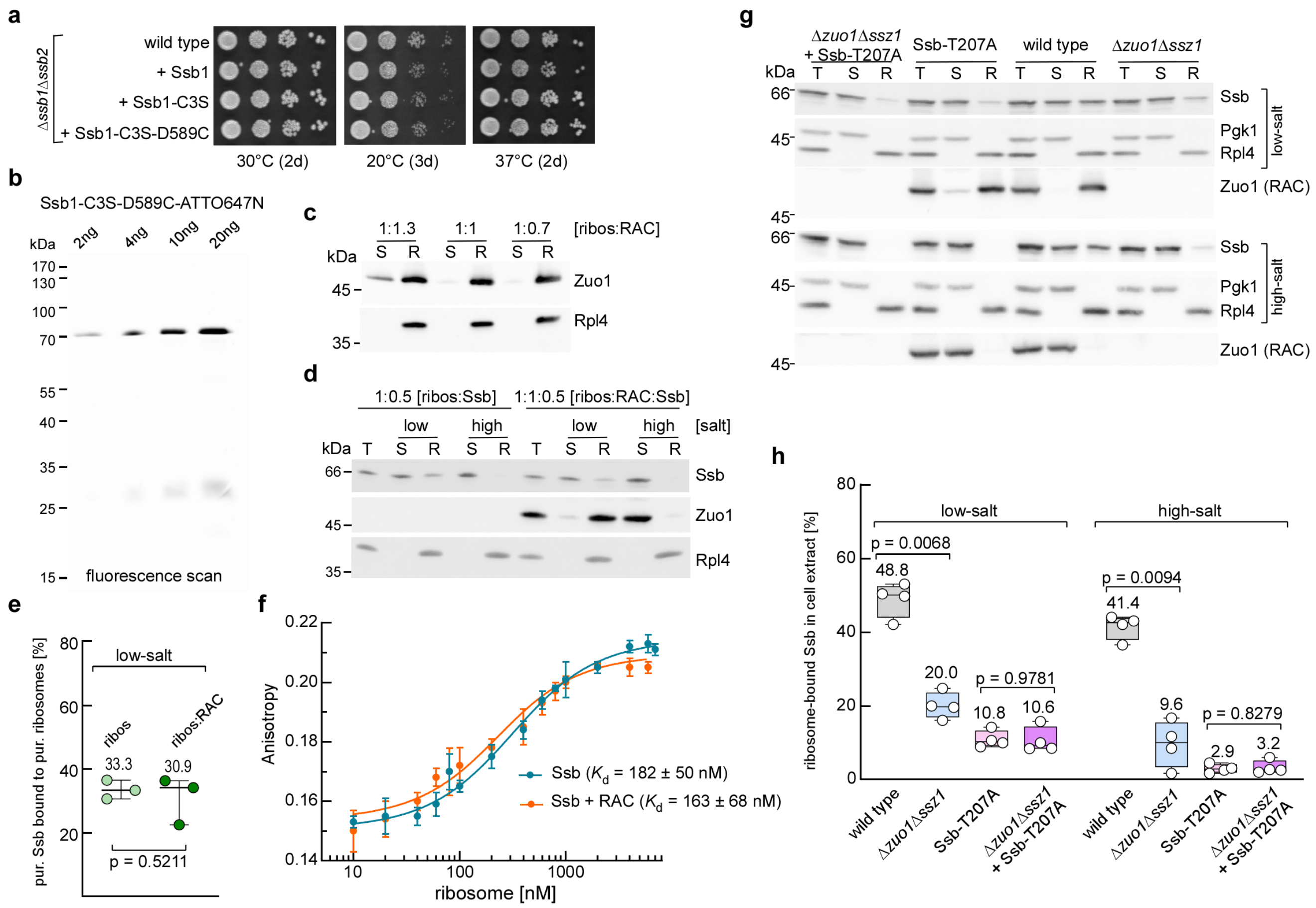

Supplementary Fig. 8. ATP-bound Ssb binds to ribosomes independently of RAC. (a) Cells expressing cysteine-free Ssb display wild type growth. Serial 10-fold dilutions of logarithmically growing Δ*ssb1*Δ*ssb2* expressing plasmid-encoded wild type Ssb1, Ssb1-C3S (C454S-C435S-C20S), or Ssb1-C3S-D589C (C454S-C435S-C20S-D589C) were spotted onto YPD plates, which were incubated for the indicated times at the indicated temperatures. **(b)** Fluorescence scan of Ssb-ATTO647N. Fluorescent scan of a Tris-Tricine gel loaded with 2 -20 ng of Ssb1-C3S-D589C-ATTO647N. **(c)** RAC forms a 1:1 complex with purified non-translating ribosomes. Ribosome-binding assays were performed in low-salt conditions as described in Supplementary Fig. 6c. RAC was detected in the supernatant only, when applied in molar excess over ribosomes. Supernatant (S), ribosomal pellet (R) **(d)** RAC does not affect binding of Ssb to purified ribosomes. Purified Ssb was incubated with pre-formed 1:1 ribosome●RAC complexes (see panel **c**) and ribosome-binding assays were performed in low-salt or high-salt conditions as described in Supplementary Fig. 6c. Supernatants (S) and ribosomal pellets (R) were analyzed by immunoblotting with antibodies directed against the indicated proteins. **(e)** Statistical analysis of Ssb ribosome-binding to non-translating ribosomes and ribosome●RAC complexes. Paired t test with data from 3 independent experiments (dots) as examplified in **d**; lines: minimum, mean, and maximum; and values: means. **(f)** RAC does not enhance the affinity of Ssb for empty ribosomes. Fluorescence anisotropy-based binding assays were performed with Ssb-ATTO647N and purified non-translating 80S ribosomes and pre-formed ribosome●RAC complexes. Shown are Anisotropy binding curves of a single biological replicate, the center indicates the mean, error bars represent the standard deviation of five technical replicates. Dissociation constants (K_d) were determined with a 1:1 binding model based on 8 (Ssb, same as shown in Fig. 4a) or 3 (Ssb + RAC) biological replicates. **(g)** Ribosome-binding of the ATPase deficient Ssb-T207A mutant is RAC-independent. Ribosome-binding assays were performed with total cell extract derived from wild type, Ssb-T207A, Δ*zuo1*Δ*ssz1*, or Δ*zuo1*Δ*ssz1* Ssb-T207A cells in low-salt (upper panel) or high-salt (lower panel) conditions as described in Supplementary Fig. 6c. Samples were analyzed by immunoblotting with antibodies directed against the indicated proteins. **(h)** Two-way ANOVA was performed with data from four independent experiments (dots), as exemplified in panel **g**. Boxes indicate the 25th to 75th percentiles; lines represent the minimum, median, and maximum values; values shown are means.

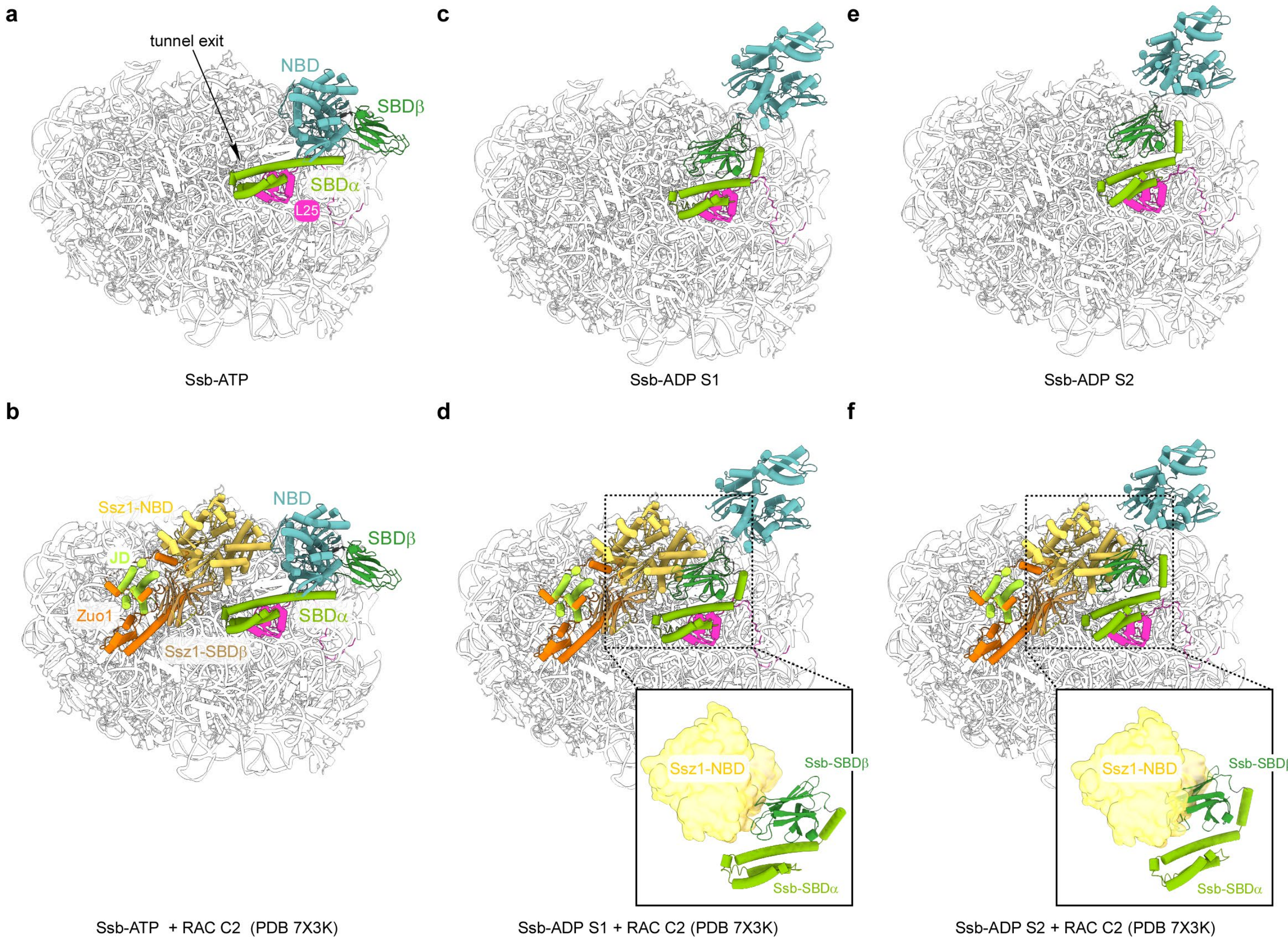

Supplementary Fig. 9. Superimposition of Ssb-ATP, Ssb-ADP S1, and Ssb-ADP S2 with RAC. Ribosomal 60S subunit [10] (PDB 6T7I) superimposed with (**a**) Ssb-ATP, aligned according to the interaction of Ssb-SBDα in the Ssb-ADP S1 state (Methods and Supplementary Note 2); (**b**) Ssb-ATP as in **a** and RAC C2 (PDB 7X3K) [11]; **(c)** the atomic model of Ssb-ADP S1 (Fig. 1b); (**d**) Ssb-ADP S1 and RAC C2; (**e**) the atomic model of Ssb-ADP S2 (Fig. 1b); (**f**) Ssb-ADP S2 and RAC C2. The inset in **d** highlights close proximity between the Ssz1-NBD and the Ssb-SBDβ in Ssb-ADP S1, the inset in **f** highlights the clash between the Ssz1-NBD and the Ssb-SBDβ in Ssb-ADP S2. Full-length Ssb-ADP and Ssb-ATP models including the NBD were generated with I-TASSER (see Methods). Ribosomal 60S subunit (light gray), Rpl25 (light magenta), Zuo1 (orange), Zuo1-LP (yellow), Zuo1-JD (limon), Ssz1-NBD (yellow orange), Ssz1-SBDβ (sand), Ssb-NBD (light teal), Ssb-SBDβ (forest) Ssb-SBDα (split pea)

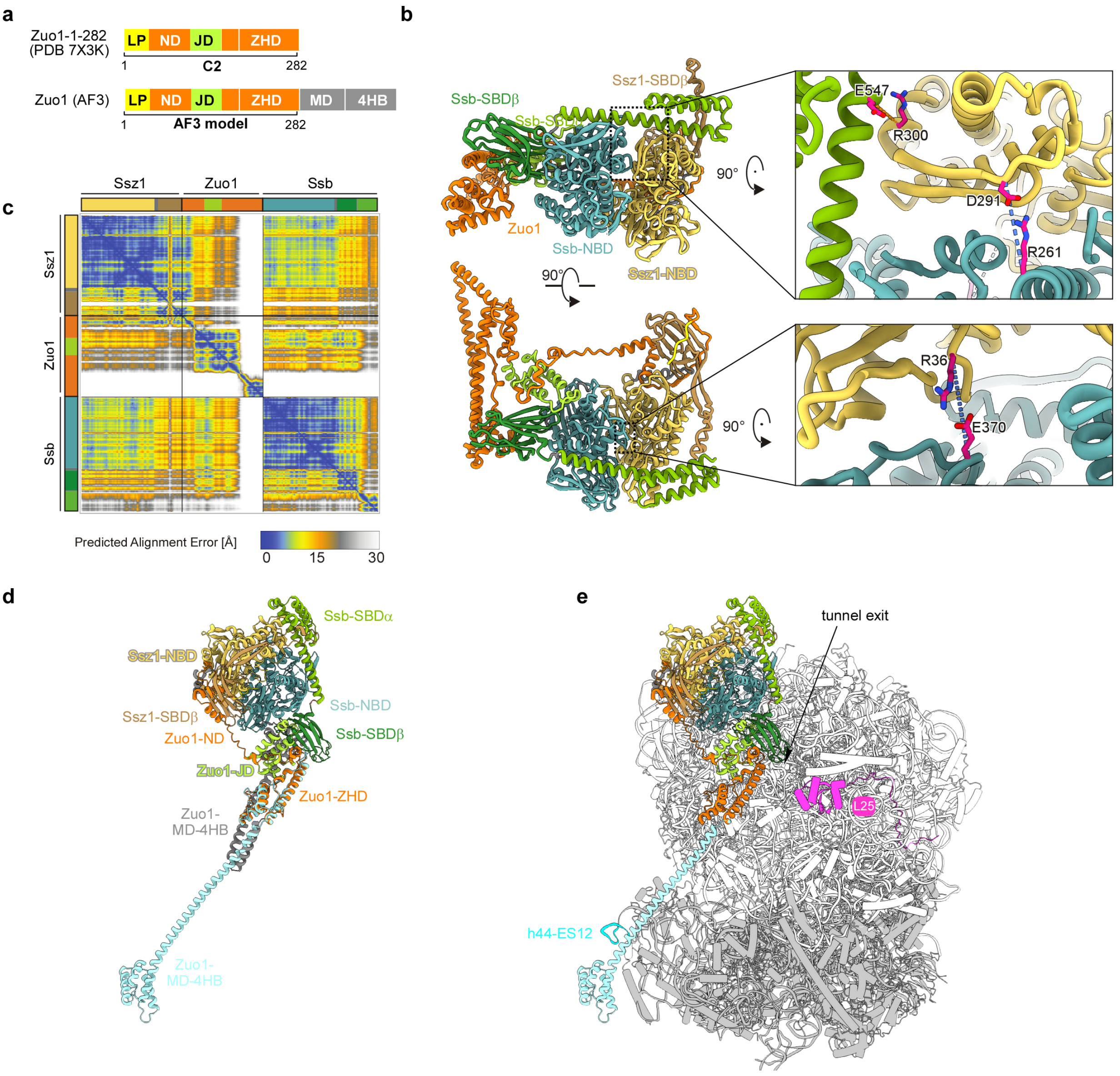

Supplementary Fig. 10. Structural details of the Ssb-ATP•RAC complex as predicted by AlphaFold 3 (AF3). (**a**) Zuo1 domains depicted in the RAC-Ssb cycle (Fig. 5 and Supplementary Movies 1-5). Upper domain map: Zuo1-1-282 as resolved in PDB 7X3K [11]. Note that PDB 7X3K provides an atomic model of Zuo1-1-282 in complex with full-length Ssz1. Lower domain map: indicates that Zuo1 from the Ssb-ATP•RAC model was truncated to the same length for consistency. For details, refer to Supplementary Note 2. (**b**) The Ssb-NBD and Ssz1-NBD associate with each other in the AF3-prediced Ssb-ATP•RAC complex. Insets show close-ups of the Ssb-NBD and Ssz1-NBD interface. The lines between highlighted residues are colored according to their predicted aligned error (PAE) as in **c**. Residues Ssb-R261 ↔ Ssz1-D291 (2.9 Å), Ssb-E370 ↔ Ssz1-R36 (2.6 Å), and Ssb-E547 ↔ Ssz1-R300 (5.8 Å). The distances in the AF3 model are consistent with salt-bridge formation and are supported by previous analysis of the corresponding triple charge-reversal mutations in Ssb and Ssz1 [6] and by the observation that the combination of the Ssb-R261D-E370R-E547R mutations (termed Ssb-3*) with the Ssb-DD1-DD2 mutations (Fig. 3a) synergistically reduced ribosome-binding of Ssb (Fig. 3h). (**c**) Predicted Aligned Error (PAE) plot of the Ssb-ATP•RAC model. The tilted Zuo1-MD-4HB is predicted with low confidence. (**d**) Superposition of the Ssb-ATP•RAC model with the atomic model of Zuo1-MD-4HB [12] (PDB 7Z3O, light cyan). The tilted Zuo1-MD-4HB of the AF3 prediction is shown in gray. (**e**) The Ssb-ATP•RAC model is compatible with association of the Zuo1-4HB with 18S rRNA h44-ES12 (cyan). Superposition of a stalled ribosome [10] (PDB 6T7I) with the Ssb-ATP•RAC/PDB 7Z3O chimera as shown in **d**. Zuo1 LP-motif (LP, yellow), N-domain (ND, orange), J-domain (JD, limon), zuotin homology domain (ZHD, orange), middle domain (MD, orange), 4-helix bundle (4HB, orange) Ssz1-NBD (yellow orange), Ssz1-SBDβ (sand), Ssb-NBD (light teal), Ssb-SBDβ (forest) Ssb-SBDα (split pea).

Supplementary Notes

Supplementary Note 1. Positioning of Ssb in the atomic models Ssb-ADP S1, Ssb-ADP S2, and in the superimposed Ssb-ATP model. Two major ribosomal contacts of Ssb are shared among the atomic models Ssb-ADP S1, Ssb-ADP S2, and the superimposed model of ribosome-bound Ssb-ATP. First, the electrostatic interactions between helix Ssb-αD (RKKR-motif) and Rpl25 (EDD-motif) in Ssb-ADP S1 (Fig. 2a), Ssb-ADP S2 (Supplementary Fig. 6a), and Ssb-ATP (Supplementary Fig. 7c). These contacts were verified by biochemical analysis (Figs. 2,3). Second, the contact formed between Ssb-K567/Ssb-R568 in the loop connecting Ssb-αB and Ssb-αC (Supplementary Fig. 5c) and 25S rRNA h59-ES24 (Supplementary Fig. 5a,b and Supplementary Fig. 7c). The latter contact is consistent with previous *in vivo* crosslinking and analysis of cDNA (CRAC) data [5], and with the observation that ribosome-binding of Ssb carrying the K567E/R568E mutations is reduced by more than 50% [5]. In the Ssb-ADP S2 atomic model, helix Ssb-αA of SBDα (Supplementary Fig. 5c) and the entire Ssb-SBDβ are positioned closer to the ribosomal surface than in Ssb-ADP S1 and the Ssb-ATP models. Specifically, Ssb-ADP S2 is in close proximity to Rpl35, Rpl19, and Rpl26, whereas Ssb-ADP S1 is not (Supplementary Figs. 5a,b). This suggests that (i) *in vitro* crosslinking obtained with the amino-specific crosslinker BS^3 (spacer length of ~11 Å) between Ssb and Rpl35/Rpl19 [5], and (ii) *in vivo* photo-crosslinking (spacer length of ~12 Å, [13]) of Ssb-R545 to Rpl35, Ssb-K497 to Rpl26, and Rpl26-K84, Rpl26-E88, Rpl35-E27, Rpl35-R38 to Ssb [6] originate from ribosomes with Ssb-ADP bound in the S2 state, or in a similar conformation (Supplementary Figs. 5a). Moreover, residues 428-430 (KRR-motif) in Ssb-SBDβ are close to 5.8S rRNA nucleotides *UUC* (81-83) in the atomic model of Ssb-ADP S2, but not in S1 (Supplementary Fig. 5a,b back view). Previous mutational analysis revealed that replacement of the KRR-motif with alanine residues moderately reduced ribosome-binding of Ssb [7], suggesting that Ssb-ADP S2 binds the ribosome more stably than Ssb-ADP S1.

Supplementary Note 2. Models for the RAC-Ssb chaperone cycle depicted in Fig. 5. For the model in Fig. 5 stalled ribosomes [10] (PDB 6T7I) were superimposed with different RAC/Ssb atomic models (cyro-EM) or structural models (predicted or superimposed). Ribosome-bound Ssb-ATP (Fig. 5, stages 2 and 3) corresponds to the structural model shown in Supplementary Fig. 7c. Ribosome-bound RAC (Fig. 5, stages 3 and 5) corresponds to the atomic model PDB 7X3K [11], which comprises full-length *S.c.* Ssz1 and Zuo1-1-282 (Supplementary Fig. 10a). Ssb-ATP●RAC (Fig. 5, stage 4) is based on an AF3-predicted model [14] of *S.c.* Ssb1, Ssz1, and Zuo1 (Supplementary Fig. 10a-d), which exhibits the following characteristics: (i) Ssb is in the open, ATP-bound conformation, which closely resembles PDB 5TKY [5]; (ii) the Ssb-NBD interacts with the Ssz1-NBD via electrostatic interactions which are consistent with previous *in vivo* crosslinking data [6] and the data shown in Fig. 3g,h (Supplementary Fig. 10b); (iii) the conformations of Ssz1, Zuo1-JD, and Zuo1-ZHD (residues 95–300) closely resemble those in PDB 7Z3O [12], but the Zuo1-ND domain differs, appearing extended in the AF3 model (Supplementary Fig. 10b,d,e); (iv) the Zuo1-JD is at the interface of Ssb-NBD and Ssb-SBD, poised to induce ATP hydrolysis by Ssb (Supplementary Fig. 10b,d); (v) the Zuo1-MD-4HB, predicted with low confidence (Supplementary Fig. 10c), is incompatible with ribosome-binding of the Ssb-ATP●RAC complex. Zuo1-MD-4HB (residues 283-443, Supplementary Fig. 10a) was therefore removed from the AF3 model (Fig. 5 stage 4 and Supplementary Movie 3). We found that superimposition of Zuo1-171–305 with the Zuo1-MD-4HB domains (*Chaetomium thermophilum* residues 176-446) from PDB 7Z3O [12] generates a chimera that places the Zuo1-4HB in close proximity to h44-ES12 of 18S rRNA (Supplementary Fig. 10d,e). The AF3-predicted Ssb-ATP●RAC model is thus consistent with the previously reported binding of the Zuo1-4HB to 18S rRNA h44-ES12 [15,16]. Ribosome-bound Ssb-ADP corresponds to the atomic models depicted in Fig. 1b. Specifically, Ssb-ADP S1 was used for Fig. 5, stage 5, while Ssb-ADP S2 was used for Fig. 5, stage 6.

Supplementary Tables

Supplementary Table 1. Data collection and Processing.

	Ssb-ADP S1	Ssb-ADP S2
EMD ID	EMD-53860	EMD-53861
Microscope	Titan Krios G4i	
Voltage [kV]	300	
Camera	Falcon 4i	
Magnification	130,000 x	
Pixel size at detector [Å/px^2]	0.951	
Total electron exposure [e-/Å^2]	40	
EER number of fractions [No.]	40	
Defocus range [µm]	-1.0 to -2.0	
Energy filter slit width [eV]	10	
Automation software	Thermo Fisher Scientific EPU	
Micrographs collected [No.]	54.286	
Total extracted particles [No.]	3.512.138	
Final particle images [No.]	32.921	14.049
Point-group symmetry	C1	
Resolution global [Å]	2.8	3.0
FSC-threshold	0.143	
Resolution range [Å]	2.7 to 8.0	2.7 to 10.3
**Refinement**		
PDB ID	PDB 9R9O	PDB 9R9P
Initial Model used	PDB 6T7I, AF3, AF2	
Model-resolution	2.9	3.0
FSC-threshold	0.5	
Map sharpening B-factor [Å^2]	n.a.	n.a.
Model composition		
Non-hydrogen atoms	194.787	
Protein residues	10.994	
Nucleotides	5.067	
B factors [Å^2] (min/max/mean)		
Protein	19.01/216.71/61.53	26.88/263.70/67.30
Nucleotides	23.80/241.63/61.68	25.36/279.76/73.39
R.m.s deviations		
Bond length [Å]	0.009	0.009
Bond angle [°]	0.655	0.691
Validation		
MolProbability Score	1.42	1.61
Clashscore	2.38	3.27
Poor rotamers [%]	1.59	1.71
Ramachandran Plot		
Favored [%]	96.42	95.55
Allowed [%]	3.55	4.42
Disallowed [%]	0.03	0.03

Supplementary Table 2. Plasmids for protein expression in yeast and *E. coli.*

plasmid	resistence marker (*E. coli*) ori and marker gene (yeast)	
pYEplac112-Rpl25	Amp^{R}, 2µ, *TRP1*	this study
pYEplac112-Rpl25-AAA	Amp^{R}, 2µ, *TRP1*	this study
pYEplac112-Rpl25-KKK	Amp^{R}, 2µ, *TRP1*	this study
pSP65-Ssb1	Amp^{R}	this study
pSP65-Ssb1-C454S-C435S-C20S	Amp^{R}	this study
pYCplac33-Ssb1	Amp^{R}, CEN, *URA3*	[17]
pYCplac33-Ssb1-C3S	Amp^{R}, CEN, *URA3*	this study
pYCplac33-Ssb1-C3S-D589C	Amp^{R}, CEN, *URA3*	this study
pYCplac33-Ssb1-DD1-DD2	Amp^{R}, CEN, *URA3*	this study
pYCplac33-Ssb1-3*	Amp^{R}, CEN, *URA3*	this study
pYCplac33-Ssb1-3*-DD1-DD2	Amp^{R}, CEN, *URA3*	this study
pET28a-His$_6$-Ssb1-T207A	Kan^{R}	this study
pET28a-His$_6$-Ssb1-DD1-DD2	Kan^{R}	this study
pET28a-His$_6$-Ssb1-C3S-D589C	Kan^{R}	this study
pET28N-His$_6$-Ssz1	Kan^{R}	this study
pETcoco2-Zuo1	Amp^{R}	[17]
pET28a-His$_6$-Ssb1	Kan^{R}	[18]
pET28a- His$_6$-Snl1-ΔTM	Kan^{R}	this study
pSPUTK-FLAG-Pgk1	Amp^{R}	[19]
pSPUTK-Pgk1	Amp^{R}	[19]
pSPUTK-Dap2	Amp^{R}	[19]
pRCC-K_SSB1/SSB2_PAM634	Amp^{R}, Kan^{R}	this study
pRCC-K SSB1_PAM1789	Amp^{R}, Kan^{R}	this study
pRCC-K_SSB1/SSB2_PAM1823	Amp^{R}, Kan^{R}	this study
pYEplac195-Dap2-3HA	Amp^{R}, 2µ, *URA3*	this study
pRCC-N_SSB2_PAM1789	Amp^{R}, Nourseothricin^{R} ,	this study

Supplementary Table 3. Yeast strains.

yeast strains	genotype	reference
MH272-3fα	*ura3 leu2 his3 trp1 ade2*	[20]
MH272-3fa	*ura3 leu2 his3 trp1 ade2*	[20]
Δ*srp54*	*srp54::KANMX4*	[21]
*myc*Ssb1-ΔC23	*ssb1::ADE2 ssb2::HIS3* + pCM190-mycSsb1-ΔC23	[5]
*myc*Ssb1	*ssb1::ADE2 ssb2::HIS3* + pCM190-mycSsb1	[5]
Δ*ssb1*Δ*ssb2* + Ssb1	*ssb1::ADE2, ssb2:: ADE2* + pYCplac33-Ssb1	this study and [17]
Δ*ssb1*Δ*ssb2* + Ssb1-DD1-DD2	*ssb1::ADE2, ssb2::HIS3* + pYCplac33-Ssb1-DD1-DD2	this study and [22]
Δ*ssb1*Δ*ssb2* + Ssb1-3*	*ssb1::ADE2, ssb2::HIS3* + pYCplac33-Ssb1-3*	this study and [22]
Δ*ssb1*Δ*ssb2* + Ssb1-3*-DD1DD2	*ssb1::ADE2, ssb2::HIS3* + pYCplac33-Ssb1-3*-DD1-DD2	this study and [22]
Ssb1-C3S	*ssb1::ADE2, ssb2:: ADE2* + pYCplac33-Ssb1-C3S	this study
Ssb1-C3S-D589C	*ssb1::ADE2, ssb2:: ADE2* + pYCplac33-Ssb1-C3S-D589C	this study
Δ*rpl25* + Rpl25	*rpl25::KanMX4* + pYCplac112-Rpl25	this study
Rpl25-AAA	*rpl25::KanMX4* + pYCplac112-Rpl25-AAA	this study
Rpl25-KKK	*rpl25::KanMX4* + pYCplac112-Rpl25-KKK	this study
Rpl25-KKK+Dap2-3HA	*rpl25::KanMX4* + pYCplac112-Rpl25-KKK + pYEplac195-Dap2-3HA	this study
MH272-3fα + Dap2-3HA	*ura3 leu2 his3 trp1 ade2* + pYEplac195-Dap2-3HA	this study
Δ*srp54* + Dap2-3HA	*srp54::KANMX4* + pYEplac195-Dap2-3HA	this study
Ssb-DD1-DD2	*ura3 leu2 his3 trp1 ade2, ssb1-R596D-K597D-K603D-R604D, ssb2-R596D-K597D-K603D-R604D*	this study
Ssb-T207A	*ura3 leu2 his3 trp1 ade2, ssb1-T207A, ssb2-T207A*	this study
Δ*ssb1*Δ*ssb2*	*ssb1::ADE2, ssb2:: ADE2*	[17]
Δ*ssb1*Δ*ssb2*	*ssb1::ADE2, ssb2::HIS3*	[22]
Δ*zuo1*Δ*ssz1*	*zuo1::TRP1, ssz1::LEU2*	[23]
Δ*ssz1*	*ssz1::LEU2*	[23]
Δ*ssz1* + Ssb1-DD1-DD2	*ssz1::LEU2 ssb1::ADE2, ssb2::ADE2* + pYCplac33-Ssb1-DD1-DD2	this study and [17]
Δ*ssb1*Δ*ssb2*Δ*zuo1*Δ*ssz1*	*ssb1::ADE2, ssb2::ADE, zuo1::TRP1, ssz1::LEU2*	[24]
Δ*RAC* Ssb-T207A	*zuo1::TRP1, ssz1::LEU2 ssb1-T207A, ssb2-T207A*	this study

Supplementary Table 4. DNA sequences for cloning and generation of yeast deletion strains.

synthetic DNA	5'-3' sequence
RPL25-E77A-D131A-D134A	AAATATGCTTCCAAGGCTGTTCCACATTACAACAGATTGGACTCATACAAGGTCATTGAGCAACCAATCACTTCTGAAACCGCTA<br>TGAAGAAGGTTGctGATGGTAACATTTTGGTTTTCCAAGTTTCCATGAAAGCTAACAAATACCAAATCAAGAAGGCCGTCAAGGA<br>ATTATACGAAGTTGACGTATTGAAGGTTAACACTTTGGTTAGACCAAACGGTACCAAGAAGGCTTACGTTAGATTGACTGCTGAC<br>TACGctGCTTTGGctATTGCTAACAGAATCGGTTACATTTAATCTAATTGGTTTAATTAATAAATTTAAT
RPL25-E77K-D131K-D134K	AAATATGCTTCCAAGGCTGTTCCACATTACAACAGATTGGACTCATACAAGGTCATTGAGCAACCAATCACTTCTGAAACCGCTA<br>TGAAGAAGGTTAAGGATGGTAACATTTTGGTTTTCCAAGTTTCCATGAAAGCTAACAAATACCAAATCAAGAAGGCCGTCAAGGA<br>ATTATACGAAGTTGACGTATTGAAGGTTAACACTTTGGTTAGACCAAACGGTACCAAGAAGGCTTACGTTAGATTGACTGCTGAC<br>TACAAGGCTTTGAAGATTGCTAACAGAATCGGTTACATTTAATCTAATTGGTTTAATTAATAAATTTAAT
Ssb1-C454S-C435S-C20S	TTCCAAGGTGCTATCGGTATCGATTTAGGTACAACCTACTCTAGTGTTGCTACTTACGAATCCTCCGTTGAAATTATTGCCAACG<br>AACAAGGTAACAGAGTCACCCCATCTTTCGTTGCTTTCACTCCAGAAGAAAGATTGATTGGTGATGCTGCCAAGAACCAAGCTGC<br>TTTGAACCCAAGAAACACTGTCTTCGATGCTAAGCGTTTGATTGGTAGAAGATTCGACGACGAATCTGTTCAAAAGGACATGAAG<br>ACCTGGCCTTTCAAGGTTATCGACGTCGATGGTAACCCAGTCATCGAAGTCCAATACTTGGAAGAAACCAAGACTTTCTCCCCAC<br>AAGAAATTTCCGCTATGGTTTTGACCAAGATGAAGGAAATTGCTGAAGCTAAGATTGGTAAGAAGGTTGAAAAGGCCGTCATTAC<br>TGTCCCAGCTTACTTTAACGACGCTCAAAGACAAGCTACCAAGGATGCCGGTGCCATTTCTGGTTTGAACGTTTTGCGTATCATC<br>AACGAACCTACTGCCGCTGCTATTGCTTACGGTCTAGGTGCTGGTAAGTCCGAAAAGGAAAGACATGTTTTGATTTTCGATTTGG<br>GTGGTGGTACTTTCGATGTTTCCTTGTTGCACATTGCTGGTGGTGTTTACACTGTTAAATCTACTTCCGGTAACACTCACTTGGG<br>TGGTCAAGATTTCGACACCAACTTGTTGGAACACTTCAAGGCTGAATTCAAGAAGAAGACTGGTTTGGACATCTCCGACGATGCC<br>AGAGCTTTGAGAAGATTGAGAACTGCTGCTGAAAGAGCTAAGAGAACCTTATCTTCTGTCACTCAAACTACCGTTGAAGTTGACT<br>CTTTGTTTGACGGTGAAGATTTCGAATCCTCTTTGACTAGAGCTAGATTTGAAGACTTGAACGCCGCATTGTTCAAGTCTACTTT<br>GGAACCTGTTGAACAAGTTTTGAAGGATGCTAAGATCTCTAAGTCTCAAATCGACGAAGTTGTCTTGGTTGGTGGTTCCACCAGA<br>ATTCCAAAGGTCCAAAAGTTGTTGTCTGACTTCTTTGACGGTAAGCAATTGGAAAAATCTATTAACCCAGATGAAGCTGTTGCTT<br>ACGGTGCTGCTGTTCAAGGTGCTATCTTGACCGGCCAATCCACATCTGACGAAACCAAGGACTTGTTGTTGTTAGATGTTGCTCC<br>ATTATCTCTAGGTGTTGGTATGCAAGGTGACATGTTCGGTATCGTTGTTCCAAGAAACACTACTGTTCCAACCATCAAGAGAAGA<br>ACCTTTACTACAAGTGCTGACAACCAAACCACCGTTCAATTCCCAGTCTACCAAGGTGAACGTGTTAACAGTAAAGAAAACACTT<br>TGTTGGGTGAATTCGACTTGAAGAACATCCCAATGATGCCAGCTGGTGAACCAGTCTTGGAAGCTATCTTCGAAGTTGATGCTAA<br>CGGTATCTTGAAGGTTACTGCCGTCGAAAAGTCTACCGGTAAGTCTTCTAACATCACTATCTCTAACGCTGTTGGTAGATTGTCT<br>TCTGAAGAAATTGAAAAGATGGTTAACCAAGCTGAAGAGTTCAAGGCTGCCGATGAAGCTTTTGCCAAGAAGCA
Ssb1-D589C	AAGGCTGCCGATGAAGCTTTTGCCAAGAAGCACGAAGCTAGACAAAGATTGGAATCCTACGTTGCCTCCATCGAACAAACTGTCA<br>CTGACCCAGTCTTGTCTTCTAAATTGAAGAGAGGTTCCAAGTCCAAGATTGAAGCTGCTTTGTCCGATGCTTTGGCTGCTTTGCA<br>AATCGAATGTCCATCTGCTGATGAATTGAGAAAGGCTGAAGTTGGTTTGAAGAGAGTTGTCACCAAGGCCATGTCTTCTAGATAA
Ssb1-R261D-E370R-E547R	CGGTAACACTcacttgggtgGTCAAGATTTCGACACCAACTTGTTGGAACACTTCAAGGCTGAATTCAAGAAGAAGACTGGTTTG<br>GACATCTCCGACGATGCC**gac**GCTTTGAGAAGATTGAGAACTGCTGCTGAAAGAGCTAAGAGAACCTTATCTTCTGTCACTCAAA

	CTACCGTTGAAGTTGACTCTTTGTTTGACGGTGAAGATTTCGAATCCTCTTTGACTAGAGCTAGATTTGAAGACTTGAACGCCGC<br>ATTGTTCAAGTCTACTTTGGAACCTGTTGAACAAGTTTTGAAGGATGCTAAGATCTCTAAGTCTCAAATCGACGAAGTTGTCTTG<br>GTTGGTGGTTCCACCAGAATTCCAAAGGTCCAAAAGTTGTTGTCTGACTTCTTTGACGGTAAGCAATTGGAAAAATCTATTAACC<br>CAGAT**aga**GCTGTTGCTTACGGTGCTGCTGTTCAAGGTGCTATCTTGACCGGCCAATCCACATCTGACGAAACCAAGGACTTGTT<br>GTTGTTAGATGTTGCTCCATTATCTCTAGGTGTTGGTATGCAAGGTGACATGTTCGGTATCGTTGTTCCAAGAAACACTACTGTT<br>CCAACCATCAAGAGAAGAACCTTTACTACATGTGCTGACAACCAAACCACCGTTCAATTCCCAGTCTACCAAGGTGAACGTGTTA<br>ACTGTAAAGAAAACACTTTGTTGGGTGAATTCGACTTGAAGAACATCCCAATGATGCCAGCTGGTGAACCAGTCTTGGAAGCTAT<br>CTTCGAAGTTGATGCTAACGGTATCTTGAAGGTTACTGCCGTCGAAAAGTCTACCGGTAAGTCTTCTAACATCACTATCTCTAAC<br>GCTGTTGGTAGATTGTCTTCTGAAGAAATTGAAAAGATGGTTAACCAAGCTGAAGAGTTCAAGGCTGCCGATGAAGCTTTTGCCA<br>AGAAGCACGAAGCTAGACAAAGATTG**aga**TCCTACGTTGCCTCCATCGAACAAACTGTCACTGACCCAGTCTTGTCTTCTAAATT<br>GAAGAGAGGTTCCAAGTCCAAGATTGAAGCTGCTTTGTCCGATGCTTTGGCTGCTTTGCAAATCGAAGACCCATCTGCTGATGAA<br>TTGAGAAAGGCTGAAGTTGGTTTGAAGAGAGTTGTCACCAAGGCCATGTCTTCTCGttaattaaGACTGCATTCATCACATATAT<br>ATGAATATTACTTATATTGTATATAAATTTTGTTTTCTCTTAACTTTGTATCCTTAATAAAATAAAATTCACAAGTATCACAATT<br>AAAATATTTGTTTTTCTTTCGCGTAAATATTTAGCTTGATAACTGTTACTGATATGTCTGAGGAAAGTTGATCAAGACCCAATAA<br>TAATCTATATACTGGTTGAAACAAATCAGTGCCGGTAACGCTTTTTGTATCTTGAGATGGCGTATTTCTACTCCAGCATTCTAGT<br>TAAGAAAAAGTCTAAAAATGGTTTctgcagGCATGCAAGCT
Ssb1-R261D-E370R-E547R-<br>R596D-K597D-K603D-R604D	CGGTAACACTcacttgggtgGTCAAGATTTCGACACCAACTTGTTGGAACACTTCAAGGCTGAATTCAAGAAGAAGACTGGTTTG<br>GACATCTCCGACGATGCC**gac**GCTTTGAGAAGATTGAGAACTGCTGCTGAAAGAGCTAAGAGAACCTTATCTTCTGTCACTCAAA<br>CTACCGTTGAAGTTGACTCTTTGTTTGACGGTGAAGATTTCGAATCCTCTTTGACTAAGCTAGATTTGAAGACTTGAACGCCGCA<br>TTGTTCAAGTCTACTTTGGAACCTGTTGAACAAGTTTTGAAGGATGCTAAGATCTCTAAGTCTCAAATCGACGAAGTTGTCTTGG<br>TTGGTGGTTCCACCAGAATTCCAAAGGTCCAAAAGTTGTTGTCTGACTTCTTTGACGGTAAGCAATTGGAAAAATCTATTAACCC<br>AGAT**aga**GCTGTTGCTTACGGTGCTGCTGTTCAAGGTGCTATCTTGACCGGCCAATCCACATCTGACGAAACCAAGGACTTGTTG<br>TTGTTAGATGTTGCTCCATTATCTCTAGGTGTTGGTATGCAAGGTGACATGTTCGGTATCGTTGTTCCAAGAAACACTACTGTTC<br>CAACCATCAAGAGAAGAACCTTTACTACATGTGCTGACAACCAAACCACCGTTCAATTCCCAGTCTACCAAGGTGAACGTGTTAA<br>CTGTAAAGAAAACACTTTGTTGGGTGAATTCGACTTGAAGAACATCCCAATGATGCCAGCTGGTGAACCAGTCTTGGAAGCTATC<br>TTCGAAGTTGATGCTAACGGTATCTTGAAGGTTACTGCCGTCGAAAAGTCTACCGGTAAGTCTTCTAACATCACTATCTCTAACG<br>CTGTTGGTAGATTGTCTTCTGAAGAAATTGAAAAGATGGTTAACCAAGCTGAAGAGTTCAAGGCTGCCGATGAAGCTTTTGCCAA<br>GAAGCACGAAGCTAGACAAAGATTG**aga**TCCTACGTTGCCTCCATCGAACAAACTGTCACTGACCCAGTCTTGTCTTCTAAATTG<br>AAGAGAGGTTCCAAGTCCAAGATTGAAGCTGCTTTGTCCGATGCTTTGGCTGCTTTGCAAATCGAAGACCCATCTGCTGATGAAT<br>TG**gatg**A**c**GCTGAAGTTGGTTTG**g**A**tgac**GTTGTCACCAAGGCCATGTCTTCTCGttaattaaGACTGCATTCATCACATATATA<br>TGAATATTACTTATATTGTATATAAATTTTGTTTTCTCTTAACTTTGTATCCTTAATAAAATAAAATTCACAAGTATCACAATTA<br>AAATATTTGTTTTTCTTTCGCGTAAATATTTAGCTTGATAACTGTTACTGATATGTCTGAGGAAAGTTGATCAAGACCCAATAAT<br>AATCTATATACTGGTTGAAACAAATCAGTGCCGGTAACGCTTTTTGTATCTTGAGATGGCGTATTTCTACTCCAGCATTCTAGTT<br>AAGAAAAAGTCTAAAAATGGTTTctgcagGCATGCAAGCT
**primer sequences**	
Rpl25-300-PstI-F	GGCTTTAGTTCTGCAGGTTAGGCTCTCCCATTACGGAGAG
Rpl25-300-BamHI-R	ATTAAATTTATTAATTAAACCAATTAGATTAAATGTAACCGATTCTG

Rpl25-StyI-F	AAATATGCTTCCAAGGCTGTTCC
Rpl25-PacI-R	ATTAAATTTATTAATTAAACCAATTAGATTAAATGTAACCGATTCTG
Ssb1-NdeI-F1	TCTGTTTCGGCATATGGCTGAAGGTGTTTTCCAAGG
Ssb1-BamHI-R1	TCTGTTTCGGGGATCCTTAACGAGAAGACATGGCCT
Pgk1-40-R	CATCATCATAGAAGTGATCTTCTTACCGTCC
Pgk1-50-R	CATCATCATGGTTGGCAAAGCAGCAAC
Pgk1-60-R	CATCATCATTCTTGGGTGGTGTTCCAAAACG
Pgk1-70-R	CATCATCATTCTACCCAAGTGAGAAGCCAAGAC
Pgk1-80-R	CATCATCATAGAGTATTTTTCGTTTCTTTCACCGTTTGG
Pgk1-87-R	CATCATCATTTCCTTAGCAACTGGAGCCAAAG
Pgk1-100-R	CATCATCATGTCGTTCAAGAAGGTGACATCC
Pgk1-120-R	GTTTTCCATCAAAATAACGGAACCTGG
Dap2-120-R	TACGTTGTTAAATCTATGTACGAT
WGP235-pCC2_Fw	TGTTGTCTGACATTTTGAGAGTTAACACCGAAATTACCAAGGCTC
WGP234-pCC1_Rv	CTTGGTGGTGTTCGTCGTATCTCTTAATCATAGAAGCAGACAATG
CC_Ssb_PAM634 fw	GTAAACACCACCAGCAATGTGCAACAGTTTTAGAGCTAGAAATAGCAAGTTAAAATAAGG
CC_Ssb_PAM634 rev	TGTTGCACATTGCTGGTGGTGTTTACGATCATTTATCTTTCACTGCGGAG
dDNA_Ssb_T207A_HindIII	GTCCGAAAAGGAAAGACATGTTTTGATTTTCGATTTGGGTGGTGGTGCTTTCGATGTAAGCTTGTTGCACATTGCTGGTGGTGTT TACACTGTTAAATCTACTTCCGGTAACACTCAC
CC_Ssb1 PAM 1789 fwd	CGAAGACCCATCTGCTGATGAATTGAGAAGTTTTAGAGCTAGAAATAGCAAGTTAAAATAAGG
CC_Ssb1 PAM 1789 rev	TTCTCAATTCATCAGCAGATGGGTCTTCGGATCATTTATCTTTCACTGCGGAG
CC_Ssb2 PAM1789 fwd	CGAAGACCCATCCGCTGATGAGTTGAGAAGTTTTAGAGCTAGAAATAGCAAGTTAAAATAAGG
CC_SSB2 PAM1789 rev	TTCTCAACTCATCAGCGGATGGGTCTTCGGATCATTTATCTTTCACTGCGGAG
CC_Ssb1/2-PAM1823 fwd	GAAGTTGGTTTGAAGAGAGTTGTCACCAGTTTTAGAGCTAGAAATAGCAAGTTAAAATAAGG
CC_Ssb1/2 PAM 1823 rev	TGGTGACAACTCTCTTCAAACCAACTTCGATCATTTATCTTTCACTGCGGAG
dDNA Ssb2_DD1	CCGATGCTTTGGCTGCTTTGCAAATCGAAGACCCATCCGCTGATGAGTTAGATGACGCAGAAGTTGGTTTGAAGAGAGTTGTCAC CAAGGCCATGTCTTCTCGTTAAG
dDNA Ssb1/Ssb2_DD1	CCGATGCTTTGGCTGCTTTGCAAATCGAAGACCCATCTGCTGATGAATTAGATGACGCTGAAGTTGGTTTGAAGAGAGTTGTCAC CAAGGCCATGTCTTCTCGTTAAG
dDNA SSB1-2_DD1_DD2_AatII	CTGCTGATGAATTAGATGACGCTGAAGTTGGTTTGGATGACGTCGTCACCAAAGCCATGTCTTCTCGTTAAGA
Ssb1-AleI-F	CGGTAACACTCACTTGGGTGG
Ssb1-PacI-R	GAATGCAGTCTTAATTAACGAGAAGACATGGCCTTGGTG
zuo1-Dis-F	GGCCGTAGCGGGATCCGGAATTTTTATAAAATTAAACACATATA
zuo1-Dis-R	GGCGCATTGGCTGCAGCATACCGCCAAAATTTCCAATAGGTCC
SSZ1-dis-F	GGCCGTAGCGGGATCCGGAATTTTTATAAAATTAAACACATATAGGCCGTAGCGGGATCCGGAGAAAGGTAGGCTTTGTAAGCAT GG

SSZ1-dis-R	GGCGCATTGGCTGCAGATACAAAGGTGAGTATAAAGTATCTTGTT
Rpl25-500-Pst1-F (*rpl25::KanMX4*)	GGCTTTAGTTCTGCAGTCTTATCTTGTATGCCCGATATAGCAACC
Rpl25-500-BamH1-R (*rpl25::KanMX4*)	GGCTTTAGTTGGATCCCAACAAACGCGCACCATCCCTACATACACA
zuo1-Dis-F (*zuo1::TRP1)*	GGCCGTAGCGGGATCCGGAATTTTTATAAAATTAAACACATATA
zuo1-Dis-R (*zuo1::TRP1*)	GGCGCATTGGCTGCAGCATACCGCCAAAATTTCCAATAGGTCC
SSZ1-dis-F (*ssz1::LEU2*)	GGCCGTAGCGGGATCCGGAATTTTTATAAAATTAAACACATATAGGCCGTAGCGGGATCCGGAGAAAGGTAGGCTTTGTAAGCATGG
SSZ1-dis-R (*ssz1::LEU2*)	GGCGCATTGGCTGCAGATACAAAGGTGAGTATAAAGTATCTTGTT

Supplementary References

1. Mayer, M. P. & Kityk, R. Insights into the molecular mechanism of allostery in Hsp70s. *Front. Mol. Biosci.* **2,** 58 (2015).
2. Mayer, M. P. & Gierasch, L. M. Recent advances in the structural and mechanistic aspects of Hsp70 molecular chaperones. *J Biol Chem* (2018).
3. Zhang, Y., Sinning, I. & Rospert, S. Two chaperones locked in an embrace: Structure and function of the ribosome-associated complex RAC. *Nat. Struct. Mol. Biol.* **24,** 611-619 (2017).
4. Zhu, X. *et al.* Structural analysis of substrate binding by the molecular chaperone DnaK. *Science* **272,** 1606-1614 (1996).
5. Gumiero, A. *et al.* Interaction of the cotranslational Hsp70 Ssb with ribosomal proteins and rRNA depends on its lid domain. *Nat. Commun.* **7,** 1-12 (2016).
6. Lee, K. *et al.* Pathway of Hsp70 interactions at the ribosome. *Nat. Commun.* **12,** 5666 (2021).
7. Hanebuth, M. A. *et al.* Multivalent contacts of the Hsp70 Ssb contribute to its architecture on ribosomes and nascent chain interaction. *Nat. Commun.* **7,** 13695 (2016).
8. Shanmuganathan, V. *et al.* Structural and mutational analysis of the ribosome-arresting human XBP1u. *Elife* **8** (2019).
9. Yaffe, M. P. & Schatz, G. Two nuclear mutations that block mitochondrial protein import in yeast. *Proc. Natl. Acad. Sci. U S A* **81,** 4819-4823 (1984).
10. Tesina, P. *et al.* Molecular mechanism of translational stalling by inhibitory codon combinations and poly(A) tracts. *EMBO J.* **39,** e103365 (2019).
11. Chen, Y., Tsai, B., Li, N. & Gao, N. Structural remodeling of ribosome associated Hsp40-Hsp70 chaperones during co-translational folding. *Nat. Commun.* **13,** 3410 (2022).
12. Kisonaite, M. *et al.* Structural inventory of cotranslational protein folding by the eukaryotic RAC complex. *Nat. Struct. Mol. Biol.* **30,** 670-677 (2023).
13. Kolhe, J. A., Babu, N. L. & Freeman, B. C. Protocol for establishing a protein interactome based on close physical proximity to a target protein within live budding yeast. *STAR Protoc* **4,** 102663 (2023).
14. Abramson, J. *et al.* Accurate structure prediction of biomolecular interactions with AlphaFold 3. *Nature* **630,** 493-500 (2024).
15. Zhang, Y. *et al.* Structural basis for interaction of a cotranslational chaperone with the eukaryotic ribosome. *Nat. Struct. Mol. Biol.* **21,** 1042-1046 (2014).
16. Lee, K., Sharma, R., Shrestha, O. K., Bingman, C. A. & Craig, E. A. Dual interaction of the Hsp70 J-protein cochaperone Zuotin with the 40S and 60S ribosomal subunits. *Nat. Struct. Mol. Biol.* **23,** 1003-1010 (2016).
17. Conz, C. *et al.* Functional characterization of the atypical Hsp70 subunit of yeast ribosome-associated complex. *J. Biol. Chem.* **282,** 33977-33984 (2007).
18. Raue, U., Oellerer, S. & Rospert, S. Association of protein biogenesis factors at the yeast ribosomal tunnel exit is affected by the translational status and nascent polypeptide sequence. *J. Biol. Chem.* **282,** 7809-7816 (2007).
19. Berndt, U., Oellerer, S., Zhang, Y., Johnson, A. E. & Rospert, S. A signal-anchor sequence stimulates signal recognition particle binding to ribosomes from inside the exit tunnel. *Proc. Natl. Acad. Sci. U S A* **106,** 1398-1403 (2009).
20. Heitman, J., Movva, N. R., Hiestand, P. C. & Hall, M. N. FK 506-binding protein proline rotamase is a target for the immunosuppressive agent FK 506 in Saccharomyces cerevisiae. *Proc. Natl. Acad. Sci. U S A* **88,** 1948-1952 (1991).
21. Zhang, Y. *et al.* NAC functions as a modulator of SRP during the early steps of protein targeting to the ER. *Mol. Biol. Cell* **23,** 3027-3040 (2012).
22. Rakwalska, M. & Rospert, S. The Ribosome-Bound Chaperones RAC and Ssb1/2p are Required for Accurate Translation in Saccharomyces cerevisiae. *Mol. Cell. Biol.* **24,** 9186-9197 (2004).
23. Gautschi, M. *et al.* RAC, a stable ribosome-associated complex in yeast formed by the DnaK-DnaJ homologs Ssz1p and zuotin. *Proc. Natl. Acad. Sci. U S A* **98,** 3762-3767 (2001).
24. Jaiswal, H. *et al.* The chaperone network connected to human ribosome-associated complex (mRAC). *Mol. Cell. Biol.* **31,** 1160-1173 (2011).
