# Supplementary material for: The cotranslational cycle of the ribosome-bound Hsp70 homolog Ssb
Source: Nat Commun. 2026 Jan 16;17:961. doi: 10.1038/s41467-025-67685-6 (PMC12847954; doi:10.1038/s41467-025-67685-6)
Supplement: Supplementary file 2 — Description of Additional Supplementary Files [file 41467_2025_67685_MOESM2_ESM.pdf]

Description of additional Supplementary Files

Supplementary Movie Legends

Supplementary Movie 1. Animated model illustrating the binding of Ssb-ATP and RAC to translating ribosomes. The animated model depicts the proposed positioning of ribosome-bound Ssb-ATP and RAC prior to the emergence of a nascent chain (Fig. 5, stages 1-3). During these initial stages of the cycle the C-terminal helix Ssb-αD of Ssb-ATP is bound to Rpl25, Ssb-SBDβ (forest) is turned away from the ribosomal tunnel exit, the Zuo1-ND (orange) is wrapped around the Zuo1-JD (limon) and interacts with the Ssz1-NBD (yellow orange) and the Ssz1 linker. The Ssz1-SBDβ (sand) is close to the ribosomal tunnel exit, associated with the Zuo1-LP (yellow), the Ssb-NBD (light teal) and Ssz1-NBD are prepositioned for complex formation. Note that the order of Ssb-ATP and RAC binding could be reversed, as both events are independent of each other. Ribosome (PDB 6T7I, ref. 64 in the main text), RAC (PDB 7X3K, ref. 16 in the main text), and Ssb-ATP (Supplementary Fig. 7c).

Supplementary Movie 2. Animated model illustrating formation of the pre-hydrolysis Ssb-ATP●RAC complex. Animated model depicting the proposed transitions leading to the formation of the pre-hydrolysis Ssb-ATP●RAC complex (Fig. 5, stages 3-4). Release of the Zuo1-LP mobilizes the adjacent Zuo1-ND and allows Ssz1 to relocate toward Ssb. This results in the release of Ssb-αD from Rpl25 and complex formation between the NBDs of the two Hsp70s. Once formed, the complex undergoes further rotational movements that ultimately result in the pre-hydrolysis Ssb-ATP●RAC complex, in which Ssb is bound to the ribosome via its interaction with the Ssz1-NBD, Ssb-SBDβ is bound to the nascent chain and positioned close to the ribosomal tunnel exit, and the Zuo1-JD is placed to trigger ATP hydrolysis by Ssb (for a detailed view of the Ssb-ATP●RAC complex see Supplementary Movie 3). Ribosome (PDB 6T7I, ref. 64 in the main text). The Ssb-ATP●RAC complex was predicted by AF3 (Supplementary Fig. 10 and Supplementary Note 2). Biochemical and structural data support the formation of Ssb-ATP●RAC, or a similar assembly (Supplementary Fig. 10 and Supplementary Note 2, references 16, 17, 21, and 28 in the main text). The Zuo1-LP and Zuo1-ND were excluded from the animated model due to their proposed flexibility and lack of structural data during the transition. Color coding of RAC and Ssb is as in Supplementary Movie 1. For further details, see Discussion and Fig. 5.

Supplementary Movie 3. Animated view of the Ssb-ATP●RAC pre-hydrolysis complex. 3D view of the AF3 model of the Ssb-ATP●RAC pre-hydrolysis complex (Fig. 5, stage 4). The Ssb-SBDβ is positioned at the tunnel exit, bound to the nascent chain, while the Zuo1-JD is oriented to trigger ATP hydrolysis. This position requires detachment of Ssb-ATP from its ribosomal binding site on Rpl25. Precise placement of Ssb-ATP in this transient ensemble is achieved through complex formation with the Ssz1-NBD. At this stage, the Zuo1-LP has returned to its binding site on Ssz1-SBDβ, facilitating reorganization of the RAC complex upon ATP hydrolysis. Ribosome (PDB 6T7I, ref. 64 in the main text), AF3 model of Ssb-ATP, Ssz1, and Zuo1-LP-ND-JD-ZHD (Supplementary Fig. 10). Color coding of RAC and Ssb is as in Supplementary Movie 1. For further details, see Discussion and Fig. 5.

Supplementary Movie 4. Animated model illustrating the conformational dynamics of RAC and Ssb during ATP hydrolysis. The animated model depicts the proposed transitions occurring during ATP hydrolysis (Fig. 5, stages 4-5). When the Zuo1-JD reaches the catalytic position in the Ssb-ATP●RAC pre-hydrolysis complex (Supplementary Movie 3), Ssb hydrolyzes ATP and adopts the Ssb-ADP conformation. The associated conformational changes precisely return helix Ssb-αD to its distant binding site on Rpl25, while Ssb-SBDβ, anchored via the nascent chain, remains nearby yet moves in a circular path around the tunnel exit. Ribosome (PDB 6T7I, ref. 64 in the main text), Ssb-ATP●RAC (Supplementary Fig. 10, The Zuo1-LP and Zuo1-ND are excluded from the intermediate stages), RAC (PDB 7X3K, ref. 16 in the main text), Ssb-ADP S1 (Fig. 1b). Color coding of RAC and Ssb is as in Supplementary Movie 1. For further details, see Discussion and Fig. 5.

Supplementary Movie 5. Animated model illustrating RAC release. The animated model depicts the proposed mechanism of RAC release at the end of the cycle (Fig. 5, stage 5-6). Due to steric clashes that occur upon transition from Ssb-ADP S1 to Ssb-ADP S2 (Fig. 1f, Supplementary Fig. 9d,f and Fig. 4c,d). Ribosome (PDB 6T7I, ref. 64 in the main text), RAC (PDB 7X3K, ref. 16 in the main text), Ssb-ADP S2 (Fig. 1b). Color coding of RAC and Ssb is as in Supplementary Movie 1. For further details, see Discussion and Fig. 5.
