# Supplementary material for: The cotranslational cycle of the ribosome-bound Hsp70 homolog Ssb
Source: Nat Commun. 2026 Jan 16;17:961. doi: 10.1038/s41467-025-67685-6 (PMC12847954; doi:10.1038/s41467-025-67685-6)
Supplement: Supplementary file 8 — Reporting Summary [file 41467_2025_67685_MOESM8_ESM.pdf]

nature portfolio

Corresponding author(s): Sabine Rospert
David Haselbach

Last updated by author(s): Nov 24, 2025

# Reporting Summary

Nature Portfolio wishes to improve the reproducibility of the work that we publish. This form provides structure for consistency and transparency in reporting. For further information on Nature Portfolio policies, see our Editorial Policies and the Editorial Policy Checklist.

## Statistics

For all statistical analyses, confirm that the following items are present in the figure legend, table legend, main text, or Methods section.

| n/a | Confirmed | |
|---|---|---|
| ☐ | ☒ | The exact sample size (*n*) for each experimental group/condition, given as a discrete number and unit of measurement |
| ☐ | ☒ | A statement on whether measurements were taken from distinct samples or whether the same sample was measured repeatedly |
| ☐ | ☒ | The statistical test(s) used AND whether they are one- or two-sided<br>*Only common tests should be described solely by name; describe more complex techniques in the Methods section.* |
| ☒ | ☐ | A description of all covariates tested |
| ☐ | ☒ | A description of any assumptions or corrections, such as tests of normality and adjustment for multiple comparisons |
| ☐ | ☒ | A full description of the statistical parameters including central tendency (e.g. means) or other basic estimates (e.g. regression coefficient) AND variation (e.g. standard deviation) or associated estimates of uncertainty (e.g. confidence intervals) |
| ☐ | ☒ | For null hypothesis testing, the test statistic (e.g. *F*, *t*, *r*) with confidence intervals, effect sizes, degrees of freedom and *P* value noted<br>*Give P values as exact values whenever suitable.* |
| ☒ | ☐ | For Bayesian analysis, information on the choice of priors and Markov chain Monte Carlo settings |
| ☒ | ☐ | For hierarchical and complex designs, identification of the appropriate level for tests and full reporting of outcomes |
| ☒ | ☐ | Estimates of effect sizes (e.g. Cohen's *d*, Pearson's *r*), indicating how they were calculated |

*Our web collection on statistics for biologists contains articles on many of the points above.*

## Software and code

Policy information about availability of computer code

| | |
|---|---|
| Data collection | Thermo Fisher Scientific EPU (v3.2) |
| Data analysis | cryoSPARC (v 4.2 – v4.6)<br>cryoDRGN (v 3.1)<br>cryolo (v1.9)<br>Relion (v 5)<br>ChimeraX (v 1.5-1.9)<br>Isolde (v 1.6-1.9)<br>Phenix (v 1.21)<br>Coot (v 0.9.2)<br>Image J 1.54g<br>GraphPad Prism 10.1.1 |

For manuscripts utilizing custom algorithms or software that are central to the research but not yet described in published literature, software must be made available to editors and reviewers. We strongly encourage code deposition in a community repository (e.g. GitHub). See the Nature Portfolio guidelines for submitting code & software for further information.

## Data

Policy information about availability of data

All manuscripts must include a data availability statement. This statement should provide the following information, where applicable:

- Accession codes, unique identifiers, or web links for publicly available datasets
- A description of any restrictions on data availability
- For clinical datasets or third party data, please ensure that the statement adheres to our policy

All data supporting the findings of this study are available within the main manuscript or Extended Data. Biological materials (strains, plasmids, antibodies) are available from the corresponding authors on reasonable request. Motion corrected micrographs with annotated particle locations were deposited in EMPIAR with the accession number EMPIAR-12933. The map and the model for Ssb-ADP S1 (complex of 80S with nascent FLAG-Pgk1-70 and Ssb1 in the S1 state) were deposited in the EMDB and PDB with the accession numbers EMD-53860, PDB 9R9O. The map and the model for Ssb-ADP S2 (complex of 80S with nascent FLAG-Pgk1-70 and Ssb1 in the S2 state) can be found via EMD-53861 and PDB-9R9P.

## Research involving human participants, their data, or biological material

Policy information about studies with human participants or human data. See also policy information about sex, gender (identity/presentation), and sexual orientation and race, ethnicity and racism.

| | |
|---|---|
| Reporting on sex and gender | not applicable |
| Reporting on race, ethnicity, or other socially relevant groupings | not applicable |
| Population characteristics | not applicable |
| Recruitment | not applicable |
| Ethics oversight | not applicable |

Note that full information on the approval of the study protocol must also be provided in the manuscript.

# Field-specific reporting

Please select the one below that is the best fit for your research. If you are not sure, read the appropriate sections before making your selection.

☒ Life sciences ☐ Behavioural & social sciences ☐ Ecological, evolutionary & environmental sciences

For a reference copy of the document with all sections, see nature.com/documents/nr-reporting-summary-flat.pdf

# Life sciences study design

All studies must disclose on these points even when the disclosure is negative.

| | |
|---|---|
| Sample size | For experiments, which involve statistical analysis sample size is indicated in the Figures |
| Data exclusions | no data exclusion |
| Replication | experiments were performed at least twice, with high reproducibility. Statistical analysis was performed with 3 or more replicates as indicated in the Figures |
| Randomization | not applicalbe |
| Blinding | not applicable |

# Reporting for specific materials, systems and methods

We require information from authors about some types of materials, experimental systems and methods used in many studies. Here, indicate whether each material, system or method listed is relevant to your study. If you are not sure if a list item applies to your research, read the appropriate section before selecting a response.

## Materials & experimental systems

| n/a | Involved in the study |
| --- | --- |
| ☐ | ☒ Antibodies |
| ☐ | ☐ Eukaryotic cell lines |
| ☐ | ☐ Palaeontology and archaeology |
| ☐ | ☐ Animals and other organisms |
| ☐ | ☐ Clinical data |
| ☐ | ☐ Dual use research of concern |
| ☐ | ☐ Plants |

## Methods

| n/a | Involved in the study |
| --- | --- |
| ☐ | ☐ ChIP-seq |
| ☐ | ☐ Flow cytometry |
| ☐ | ☐ MRI-based neuroimaging |

# Antibodies

| | |
| --- | --- |
| Antibodies used | Rabbit polyclonal antibodies ⍰-Ssb (Zhang et al, 2020) (1:10,000, #471), ⍰-Zuo1 (Raue et al, 2007) (1:20,000, #10), ⍰-Ssz1 (Raue et al, 2007) (1:8,000, #11), ⍰-Rpl35 (Gumiero et al, 2016) (1:5,000, #99), ⍰-Srp54 (Raue et al, 2007) (1:2,000, #12), ⍰-Sse1 (Raue et al, 2007) (1:10,000, #31), ⍰-Rpl25 (Zhang et al, 2012) (1:5,000, #101), ⍰-Rpl4 (Zhang et al, 2016) (1:8,000, #167), ⍰-Rps9 (Raue et al, 2007) (1:5,000, #44), and ⍰-Pgk1 (Zhang et al, 2016) (1:10,000, #407) (Eurogentec, Rospert lab antibody collection). ⍰-His6 (1:4,000, Biorad, 154295), ⍰-FLAG-tag (1:2,000, Sigma, F1804), ⍰-HA (1:2000, Invitrogen, 26183) are purified monoclonal antibodies. Horseradish peroxidase-conjugated protein A (1:5,000, Thermo Fischer Scientific, 101023) or goat anti Mouse HRP (1:10,000, Santa Cruz, sc-2748) was employed as a secondary antibody. Numbers in brackets indicate the dilution of antibodies for immunoblotting and the antibody collection number). |
| Validation | antibodies of the Rospert antibody collection (collection numbers are indicated) were validated using yeast deletion strains or, in case of essential antigen proteins, with tagged-versions of the antigen. References for the validation are given. |

# Eukaryotic cell lines

Policy information about cell lines and Sex and Gender in Research

| | |
| --- | --- |
| Cell line source(s) | not applicable |
| Authentication | not applicable |
| Mycoplasma contamination | not applicable |
| Commonly misidentified lines (See ICLAC register) | not applicalbe |

# Palaeontology and Archaeology

| | |
| --- | --- |
| Specimen provenance | not applicable |
| Specimen deposition | not applicalbe |
| Dating methods | not applicable |

☐ Tick this box to confirm that the raw and calibrated dates are available in the paper or in Supplementary Information.

| | |
| --- | --- |
| Ethics oversight | not applicalbe |

Note that full information on the approval of the study protocol must also be provided in the manuscript.

# Animals and other research organisms

Policy information about studies involving animals; ARRIVE guidelines recommended for reporting animal research, and Sex and Gender in Research

| | |
| --- | --- |
| Laboratory animals | not applicalbe |
| Wild animals | not applicalbe |
| Reporting on sex | not applicalbe |
| Field-collected samples | not applicalbe |

Ethics oversight | not applicalbe

Note that full information on the approval of the study protocol must also be provided in the manuscript.

# Clinical data

Policy information about clinical studies

All manuscripts should comply with the ICMJE guidelines for publication of clinical research and a completed CONSORT checklist must be included with all submissions.

| | |
|---|---|
| Clinical trial registration | not applicalbe |
| Study protocol | not applicalbe |
| Data collection | not applicalbe |
| Outcomes | not applicalbe |

# Dual use research of concern

Policy information about dual use research of concern

## Hazards

Could the accidental, deliberate or reckless misuse of agents or technologies generated in the work, or the application of information presented in the manuscript, pose a threat to:

| No | Yes | |
|---|---|---|
| ☒ | ☐ | Public health |
| ☒ | ☐ | National security |
| ☒ | ☐ | Crops and/or livestock |
| ☒ | ☐ | Ecosystems |
| ☒ | ☐ | Any other significant area |

## Experiments of concern

Does the work involve any of these experiments of concern:

| No | Yes | |
|---|---|---|
| ☒ | ☐ | Demonstrate how to render a vaccine ineffective |
| ☒ | ☐ | Confer resistance to therapeutically useful antibiotics or antiviral agents |
| ☒ | ☐ | Enhance the virulence of a pathogen or render a nonpathogen virulent |
| ☒ | ☐ | Increase transmissibility of a pathogen |
| ☒ | ☐ | Alter the host range of a pathogen |
| ☒ | ☐ | Enable evasion of diagnostic/detection modalities |
| ☒ | ☐ | Enable the weaponization of a biological agent or toxin |
| ☒ | ☐ | Any other potentially harmful combination of experiments and agents |

# Plants

| | |
|---|---|
| Seed stocks | not applicalbe |
| Novel plant genotypes | not applicalbe |
| Authentication | not applicalbe |

# ChIP-seq

## Data deposition

- [ ] Confirm that both raw and final processed data have been deposited in a public database such as GEO.
- [ ] Confirm that you have deposited or provided access to graph files (e.g. BED files) for the called peaks.

| | |
|---|---|
| Data access links<br>*May remain private before publication.* | not applicalbe |
| Files in database submission | not applicalbe |
| Genome browser session<br>(e.g. UCSC) | not applicalbe |

## Methodology

| | |
|---|---|
| Replicates | not applicable |
| Sequencing depth | not applicalbe |
| Antibodies | not applicalbe |
| Peak calling parameters | not applicalbe |
| Data quality | not applicalbe |
| Software | not applicalbe |

# Flow Cytometry

## Plots

Confirm that:

- [ ] The axis labels state the marker and fluorochrome used (e.g. CD4-FITC).
- [ ] The axis scales are clearly visible. Include numbers along axes only for bottom left plot of group (a 'group' is an analysis of identical markers).
- [ ] All plots are contour plots with outliers or pseudocolor plots.
- [ ] A numerical value for number of cells or percentage (with statistics) is provided.

## Methodology

| | |
|---|---|
| Sample preparation | not applicalbe |
| Instrument | not applicalbe |
| Software | not applicalbe |
| Cell population abundance | not applicalbe |
| Gating strategy | not applicalbe |

- [ ] Tick this box to confirm that a figure exemplifying the gating strategy is provided in the Supplementary Information.

# Magnetic resonance imaging

## Experimental design

| | |
|---|---|
| Design type | not applicalbe |
| Design specifications | not applicalbe |
| Behavioral performance measures | not applicalbe |

## Acquisition

| | |
|---|---|
| Imaging type(s) | not applicalbe |
| Field strength | not applicalbe |
| Sequence & imaging parameters | not applicalbe |
| Area of acquisition | not applicalbe |

Diffusion MRI ☐ Used ☐ Not used

## Preprocessing

| | |
|---|---|
| Preprocessing software | not applicalbe |
| Normalization | not applicalbe |
| Normalization template | not applicalbe |
| Noise and artifact removal | not applicalbe |
| Volume censoring | not applicalbe |

## Statistical modeling & inference

| | |
|---|---|
| Model type and settings | not applicalbe |
| Effect(s) tested | not applicalbe |

Specify type of analysis: ☐ Whole brain ☐ ROI-based ☐ Both

| | |
|---|---|
| Statistic type for inference (See Eklund et al. 2016) | not applicalbe |
| Correction | not applicalbe |

## Models & analysis

| n/a | Involved in the study |
|---|---|
| ☒ | ☐ Functional and/or effective connectivity |
| ☒ | ☐ Graph analysis |
| ☐ | ☒ Multivariate modeling or predictive analysis |

Multivariate modeling and predictive analysis: Cryo-EM: The autoencoder algorithm of cryoDRGN was used to encode particles. Particle embeddings were clustered using UMAP algorithm within the cryoDRGN software package. Details can be found in the corresponding paper.
